# Demonstration of prion-like properties of mutant huntingtin fibrils in both in vitro and in vivo paradigms

**DOI:** 10.1007/s00401-019-01973-6

**Published:** 2019-02-20

**Authors:** Maria Masnata, Giacomo Sciacca, Alexander Maxan, Luc Bousset, Hélèna L. Denis, Florian Lauruol, Linda David, Martine Saint-Pierre, Jeffrey H. Kordower, Ronald Melki, Melanie Alpaugh, Francesca Cicchetti

**Affiliations:** 1 Centre de Recherche du CHU de Québec, Axe Neurosciences, 2705 Boulevard Laurier, Québec, QC G1V 4G2 Canada; 2 Département de Psychiatrie & Neurosciences, Université Laval, Québec, QC Canada; 3 Department of Neurological Sciences, Rush University Medical Centre, Chicago, IL USA; 4 Laboratory of Neurodegenerative Diseases, Institut François Jacob, MIRCen, CEA, CNRS, 18 Route du Panorama, 92265 Fontenay-aux-Roses Cedex, France

## Abstract

In recent years, evidence has accumulated to suggest that mutant huntingtin protein (mHTT) can spread into healthy tissue in a prion-like fashion. This theory, however, remains controversial. To fully address this concept and to understand the possible consequences of mHTT spreading to Huntington’s disease pathology, we investigated the effects of exogenous human fibrillar mHTT (Q48) and huntingtin (HTT) (Q25) N-terminal fragments in three cellular models and three distinct animal paradigms. For in vitro experiments, human neuronal cells [induced pluripotent stem cell-derived GABA neurons (iGABA) and (SH-SY5Y)] as well as human THP1-derived macrophages, were incubated with recombinant mHTT fibrils. Recombinant mHTT and HTT fibrils were taken up by all cell types, inducing cell morphology changes and death. Variations in HTT aggregation were further observed following incubation with fibrils in both THP1 and SH-SY5Y cells. For in vivo experiments, adult wild-type (WT) mice received a unilateral intracerebral cortical injection and R6/2 and WT pups were administered fibrils via bilateral intraventricular injections. In both protocols, the injection of Q48 fibrils resulted in cognitive deficits and increased anxiety-like behavior. Post-mortem analysis of adult WT mice indicated that most fibrils had been degraded/cleared from the brain by 14 months post-surgery. Despite the absence of fibrils at these later time points, a change in the staining pattern of endogenous HTT was detected. A similar change was revealed in post-mortem analysis of the R6/2 mice. These effects were specific to central administration of fibrils, as mice receiving intravenous injections were not characterized by behavioral changes. In fact, peripheral administration resulted in an immune response mounting against the fibrils. Together, the in vitro and in vivo data indicate that exogenously administered mHTT is capable of both causing and exacerbating disease pathology.

**Electronic supplementary material:**

The online version of this article (10.1007/s00401-019-01973-6) contains supplementary material, which is available to authorized users.

## Introduction

Huntington’s disease (HD) is an autosomal dominant neurodegenerative disorder that typically progresses to death over 10–30 years [51]. During the pre-manifest phase, subtle changes in personality, cognition and motor control can be observed which, over time, lead to diagnosis based on motor features of the condition. Once manifest, HD patients exhibit progressive cognitive impairments that impact activities of daily living along with psychiatric disturbances that can evolve to frank psychosis and a worsening movement disorder. In the final stages, patients become demented and bedbound [24, 51, 54, 59]. The classic neuropathological feature is a massive atrophy of the caudate and putamen which results from neuronal dysfunction and loss, especially of the medium spiny projection neurons. Progressive neuronal loss and atrophy are also observed in other areas of the brain including the deep layers of the cerebral cortex and it is likely that degeneration of cortical areas is responsible for the more critical cognitive and personality-related abnormalities seen in HD patients [44, 52]. Non-neuronal cell types are also impacted, with cell-autonomous changes in neuro-inflammatory cells such as microglia having also been described [13]. Importantly, HD is caused by a CAG repeat expansion beyond 35 in exon1 of the huntingtin gene which encodes for huntingtin (HTT), a cytoplasmic protein ubiquitously expressed and present both in humans and rodents, with a particularly high expression in the brain [47]. This leads to the production of a mutant protein (mHTT) with an expanded polyglutamine stretch [5, 59].

Many proteins that play a central role in common neurological disorders, including α-synuclein, tau, TDP-43, amyloid, and SOD, have now been described to have prion-like properties [9, 28, 49]. The concept of prions as disease causing agents was pioneered in 1982 with the seminal discovery by Stanley Prusiner that neurodegeneration in sheep and goats could result from exposure to a protein in an aggregated abnormal form, specifically to what has come to be known as a prion protein [43]. It was demonstrated that this entity is capable of spreading and seeding pathology [14]. However, to be qualified as “prion”, a protein must be capable of irreversible conversion of other normal proteins into a pathogenic form that can spread disease independently. From studying the proteins associated with neurodegenerative diseases, it has become clear that various factors influence how “infectious” a specific protein is. For example, the fibrillar form of α-synuclein is more toxic than the monomeric or oligomeric precursors [42]. Different cleavage products of proteins have differential abilities to be recruited and to subsequently seed pathology. Amyloid is the classic example of this with specific truncated forms such as amyloid-β 42 conveying far greater toxicity and infectivity than other similar structures such as amyloid-β 40 [19].

From the initial reports, mHTT spreading/seeding was indeed highly debated; however, this theory is rapidly gaining support from both in vitro and in vivo experiments [4, 15, 16, 26, 32, 33, 35–37, 45]. Despite the increased acceptance of mHTT spreading and seeding, the relevance of these phenomena in HD still raises significant scepticism; scepticism which is being further challenged by recent reports that mHTT with high seeding capacity is associated with greater neuronal toxicity in a Drosophila model of HD [4]. To shed further light on these properties and their consequences, three distinct human cell models and three animal paradigms were used in which the impact of recombinant N-terminal HTT fibrillar fragments, termed HTTExon1 throughout the manuscript, was thoroughly investigated.

## Materials and methods

### Recombinant HTTExon1Q25 and Q48 fibrils

The expression and purification of human HTTExon1 with a 25 (normal) or 48 (pathological) glutamine stretch were performed, as previously described [38]. Briefly, HTTExon1 was assembled in 20 mM Tris–HCl, pH 7.5, 150 mM KCl, 5 mM MgCl_2_, 1 mM ATP, 100 mM imidazole and 10% glycerol, at 37 °C for 24 h (without shaking). HTTExon1 fibrils were centrifuged twice at 15,000*g* for 10 min and resuspended twice in phosphate-buffered saline (PBS). The fibrils and BSA were labeled with ATTO-550 or ATTO-488 (ATTO-Tec GmbH Siegen, Germany, # AD 488-35 and # AD 550-35) *N*-hydroxysulfosuccinimide (NHS) fluorophore following the manufacturer’s instructions using a protein–dye ratio of 1:2. The labeling reactions were stopped by the addition of 1 mM Tris pH 7.5. The unreacted fluorophore was removed by a final cycle of two centrifugations at 15,000*g* for 10 min and resuspension of the pelleted fibrils in PBS. The amount of ATTO-550 or ATTO-488 incorporated was assessed by mass spectrometry. The samples were de-salted with 5% acetonitrile, 0.1% Trifluoroacetic acid (TFA) and eluted from a C18 reversed-phase Zip-Tip (Millipore, Billerica, MA, USA, Cat# ZTC18M096) in 50% acetonitrile, 0.1% TFA. Peptide samples were mixed in a ratio of 1:5–1:20 (v/v) with sinapinic acid (10 mg/mL) in 50% acetonitrile and 0.1% TFA and spotted (0.5 µL) on a stainless steel MALDI target (Opti-TOF; Applied BioSystems Foster City, CA 94404 USA, Cat# 4347686). MALDI-TOF-TOF MS spectra were acquired with a MALDI-TOF⁄TOF 5800 mass spectrometer (Applied Biosystems Foster City, CA 94404 USA) using linear mode acquisition.

Fluorescently labeled HTTExon1 fibrils were fragmented for 15 min at 30 °C in 2 mL Eppendorf tubes in a VialTweeter powered by an ultrasonic processor UIS250v (250 W, 24 kHz, Hielscher Ultrasonic, Teltow, Germany) set at 75% amplitude, 0.5 s pulses every 1 s. The nature of fibrillar HTTExon1 forms before and after fragmentation was assessed using a JEOL 1400 transmission electron microscope following adsorption onto carbon-coated 200-mesh grids and negative staining with 1% uranyl acetate. The fragmented fibrils were flash frozen in liquid nitrogen and stored at − 80 °C until use.

### Cell lines and culture conditions

Experiments were performed in three different cell types: SH-SY5Y human neuroblastoma cell line, THP1 monocytes differentiated into macrophages, and iGABA high-purity post-mitotic human neural cells derived from induced pluripotent stem cells (iPSC). The concentration of fibrils was selected based on a dose response curve of SH-SY5Y cells using MTT reduction as an indicator (Online Resource 1c). The dose used in our experiments (0.005 µg/μL, e.g., 312 nM) was selected such that we observed sufficient toxicity without detrimental effects incompatible with long-term observations.

#### Incubation with fibrils

**Fig. 1:**
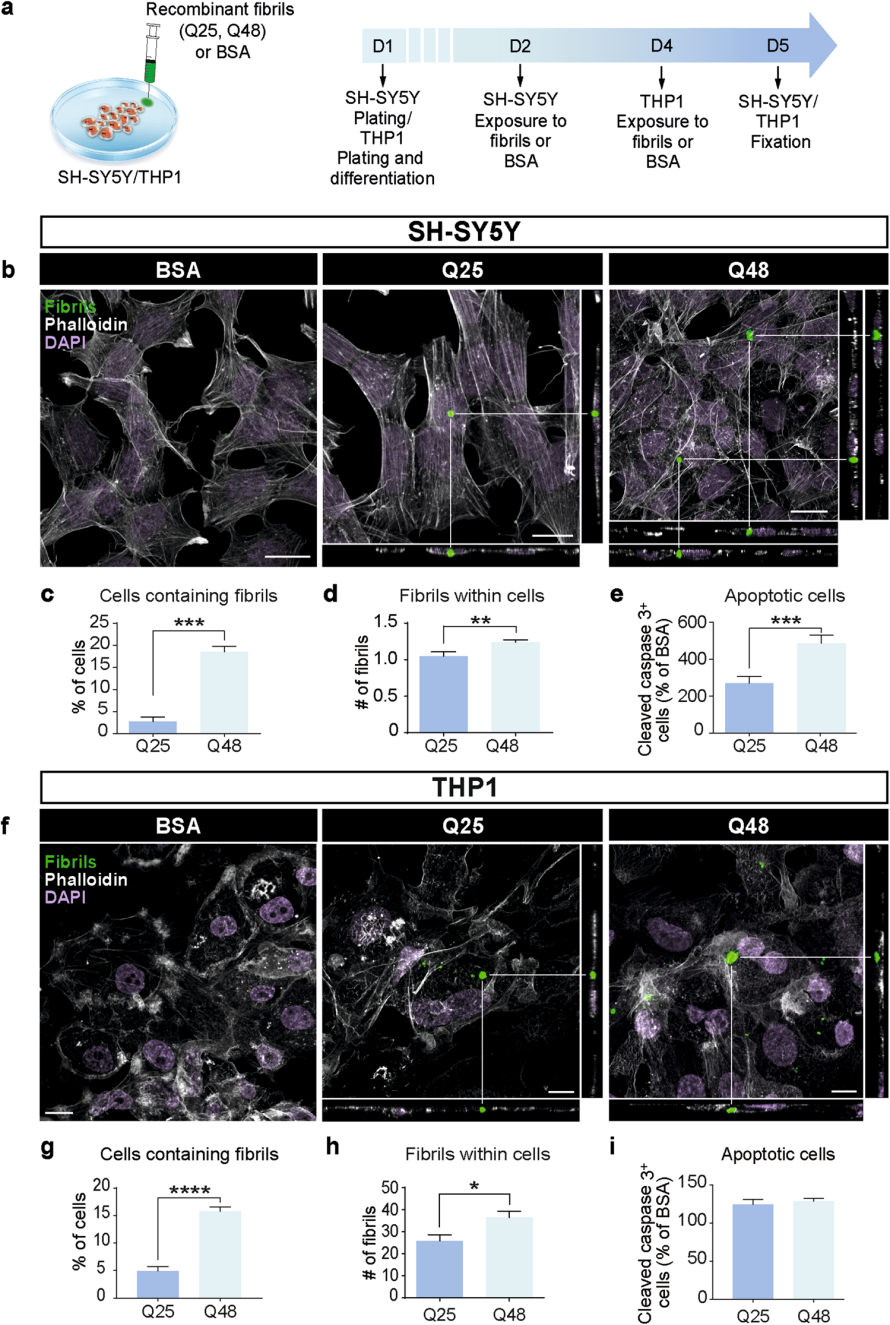
Uptake of fibrillar HTTExon1Q25 and Q48 by different cell types. Schematic of experimental design (**a**). Representative confocal photomicrographs of human SH-SY5Y cells (**b**) and THP1-derived macrophages (**f**) demonstrating uptake of both ATTO488-labeled HTTExon1Q25 and Q48 fibrils (green), 72 (SH-SY5Y) and 24 h (THP1) post-exposure, respectively. For all photomicrographs, the cell membrane was labelled with phalloidin (white) and cell nuclei were stained with DAPI (purple). The percentage of SH-SY5Y (**c**) and THP1 (**g**) cells containing puncta, the number of puncta per SH-SY5Y (**d**) and THP1 (**h**) cell and the number of apoptotic SH-SY5Y (**e**) and THP1 (**i**) cells were all calculated. All graphs are the average of three independent experiments. Data are expressed as mean ± SEM. Statistical analysis was performed using a students’ unpaired *t* test. *p < 0.05, **p < 0.01, ***p < 0.001, ****p < 0.0001. Scale bars **b** = 25 µm, **f** = 10 µm. *BSA* bovine serum albumin

The SH-SY5Y (human neuroblastoma) cell line was cultured in Dulbecco’s Modified Eagle’s Media (DMEM) F12 (Sigma-Aldrich, ON, Canada, Cat# 51445C-1000 mL) supplemented with 10% FBS (Sigma-Aldrich, Cat# F2442—500 mL) and 1X antibiotic antimycotic solution (100 units penicillin, 0.1 mg streptomycin and 0.25 µg amphotericin B per mL) (Sigma-Aldrich, A5955—100 mL). Cells were plated on gelatin-(Sigma-Aldrich, Cat# G1393) coated 12-mm coverslips (Fisher Scientific, ON, Canada, Cat# 12-545-81) in 24-well plates (Sarstedt, Numbrecht, Germany, Cat# 83.3922.005). Twenty-four hours post-plating, cells were exposed to 1 µL of 5 µg/μL mHTTExon1 fibrils (312 nM) or 1 µL of 1 µg/μL BSA (15 µM) (BSA, BioShop Canada, ON, CA, Cat# ALB999) for 3 days [38] (Fig. 1a).

The THP1 (human leukemic monocyte) cell line was cultured in DMEM (Sigma-Aldrich, ON, Canada, Cat# 56499C-50 L) supplemented with 10% FBS (Sigma-Aldrich) and 1X antibiotic/antimycotic solution (100 units penicillin, 0.1 mg streptomycin and 0.25 µg amphotericin B per mL). Cells were plated onto uncoated 12-mm coverslips (Fisher Scientific) in 24-well plates (Sarstedt) and differentiated into macrophages by adding 100 ng/mL phorbol-12-myristate-13-acetate (PMA, Sigma-Aldrich, Cat# P8139—1 mg) for a period of 72 h. Cells were then exposed to 1 µL of 5 µg/μL mHTTExon1 fibrils (312 nM) or 1 µL of 1 µg/μL BSA (15 µM) for 24 h (Fig. 1a).

The iCell Neurons (iGABA; Cellular Dynamic International, WI, USA) was thawed following company guidelines (iCell Neurons User’s Guide, Cellular Dynamic International). Briefly, cells were plated in complete maintenance medium (Cellular Dynamic International) on poly-l-ornithine-(Sigma-Aldrich, Cat# P4957—50 mL)/laminin (Sigma-Aldrich, Cat# L9393—100 UL) coated 18-mm coverslips (Menzel-Glaser, Dublin, Ireland, Cat# BB018018A1) in 12-well plates (Sarstedt, Numbrecht, Germany, Cat# 83.3921.005). The iGABA cells were treated 24 h post-plating with 1 µL of 5 µg/µL mHTTExon1 fibrils Q25 and Q48 for 3 days and untreated cells were used as control (Online Resource 1i).

#### Immunofluorescence for in vitro experiments

After treatment, SH-SY5Y cells were washed with 37 °C DMEM F12 (Sigma-Aldrich) and 1X Dulbecco’s phosphate buffered saline (DPBS) calcium magnesium (Thermo Fisher Scientific, ON, Canada, Cat# 14040133) and fixed for 20 min with 4% paraformaldehyde (PFA, Electron Microscopy Science, PA, USA, Cat# 19208) pH 7.3. Cells were washed (3 × 5 min) with 1X DPBS, permeabilized for 10 min with 0.5% Triton X-100 (Sigma-Aldrich, Cat# T8787—100 mL) in 1X PBS, and blocked for 1 h in 3% normal donkey serum (Sigma-Aldrich, Cat# D9663—10 mL) in 1X PBS. Cells were then incubated with primary anti-cleaved caspase 3 antibody (Asp 175) (1:400, Cell Signaling, ON, CA, Cat# 9661S,) or anti-HTT (1:500, Millipore, CA, USA, MAB2168,) diluted in blocking solution overnight at 4 °C, washed (3 × 5 min) in 1X DPBS, and incubated with Alexa 647 or Alexa 488 conjugated secondary antibody (1:500; Thermo Fisher Scientific, Cat# A32733 or A32723) diluted in blocking solution for 1 h at room temperature (RT). Cells were then stained with phalloidin Alexa 647 or Alexa 488 (5 µL per unit, Thermo Fisher Scientific, Cat# A22287 or A12379) for 15 min, washed (3 × 5 min) and finally incubated for 1 min with 4′,6-diamidino-2-phenylindole (DAPI) nuclear stain (0.022%, Thermo Fisher Scientific, Cat# D1306). Both Phalloidin and DAPI were diluted in 1X DPBS. Coverslips were mounted on glass slides in Fluoromount G (Thermo Fisher Scientific, Cat# 00-4958-02). After treatment, THP1 cells were washed with PBS (3 × 5 min) and fixed for 15 min with 4% PFA pH 7.3. Cells were washed with PBS (3 × 5 min), permeabilized for 4 min with 0.1% Triton X-100 (Sigma-Aldrich) and 1% BSA in PBS, washed with PBS (3 × 5 min) and blocked for 45 min in 1% BSA in PBS. Cells were then incubated with anti-cleaved caspase 3 (Asp 175) (1:400) or anti-HTT (1:500) primary antibodies diluted in blocking solution overnight at 4 °C, washed with PBS (3 × 5 min), and incubated in Alexa 647 or Alexa 546 (1:500) conjugated secondary antibodies diluted in blocking solution for 1 h at RT. Cells were then stained with DAPI and phalloidin as described for SH-SY5Y cells.

#### Filter retardation assay

SH-SY5Y cells and THP1 cells were cultured as described above and plated in 6-well plates (Sarstedt, Cat# 83.3920.005). Twenty-four hours post-plating, cells were incubated with 1 µL of 5 µg/μL mHTTExon1 fibrils [38] or 1 µL of 1 mg/mL BSA for 5 days for SH-SY5Y and 24 h for THP1 cells. Cell lysis was performed on ice, after one wash with PBS at RT, adding 150 µL of RIPA buffer (1% SDS) with 1% protease and phosphatase inhibitor cocktail 100× (Thermo Scientific, Cat# 78440). Samples were then sonicated for 2 × 10 s and resuspended with a 26 G needle five times. After 30 min on ice, samples were centrifuged at 12,000*g* for 5 min at 4 °C. The filter trap assay was performed in triplicate with 50 µg of cell lysate diluted in PBS to complete a volume of 70 µL to which 30 µL of SDS:DTT mix was added (final concentration SDS: 2%, final concentration DTT: 100 mM). Samples were boiled at 100 °C for 10 min, cooled to RT and the cellulose acetate membrane (Steriltech, WA, USA, Cat#1480025) was washed 2 × 5 min in 1% SDS in PBS prior to loading. After assembling the apparatus (HYBRI DOT Manifold, BRL Bethesda Research Laboratories, USA, Cat# 1050MM), 100 µL of sample was loaded per well. After sample filtration, the membrane was washed 2 × 5 min with 0.1% SDS in PBS in the filtration apparatus, removed from the apparatus, dried for 30 min, washed 3 × 5 min with 0.1% SDS in PBS and rinsed once in PBS to remove excess SDS. The membrane was then blocked with 5% BSA in PBS for 1 h at RT, then incubated with anti-HTT (1:500, Millipore, MAB2166) or anti-HTT clone EM48 (1:500, Millipore, MAB5374) in 2.5% BSA in PBS-Tween overnight at 4 °C. Incubation in secondary Azure spectra 800 goat anti-mouse antibody (Azure Biosystem, CA, USA, Cat# AC2135) was performed for 45 min and membranes were visualized using Odyssey CLx imaging system (LI-COR, Bad Homburg, Germany).

#### In vitro quantification

**Fig. 2:**
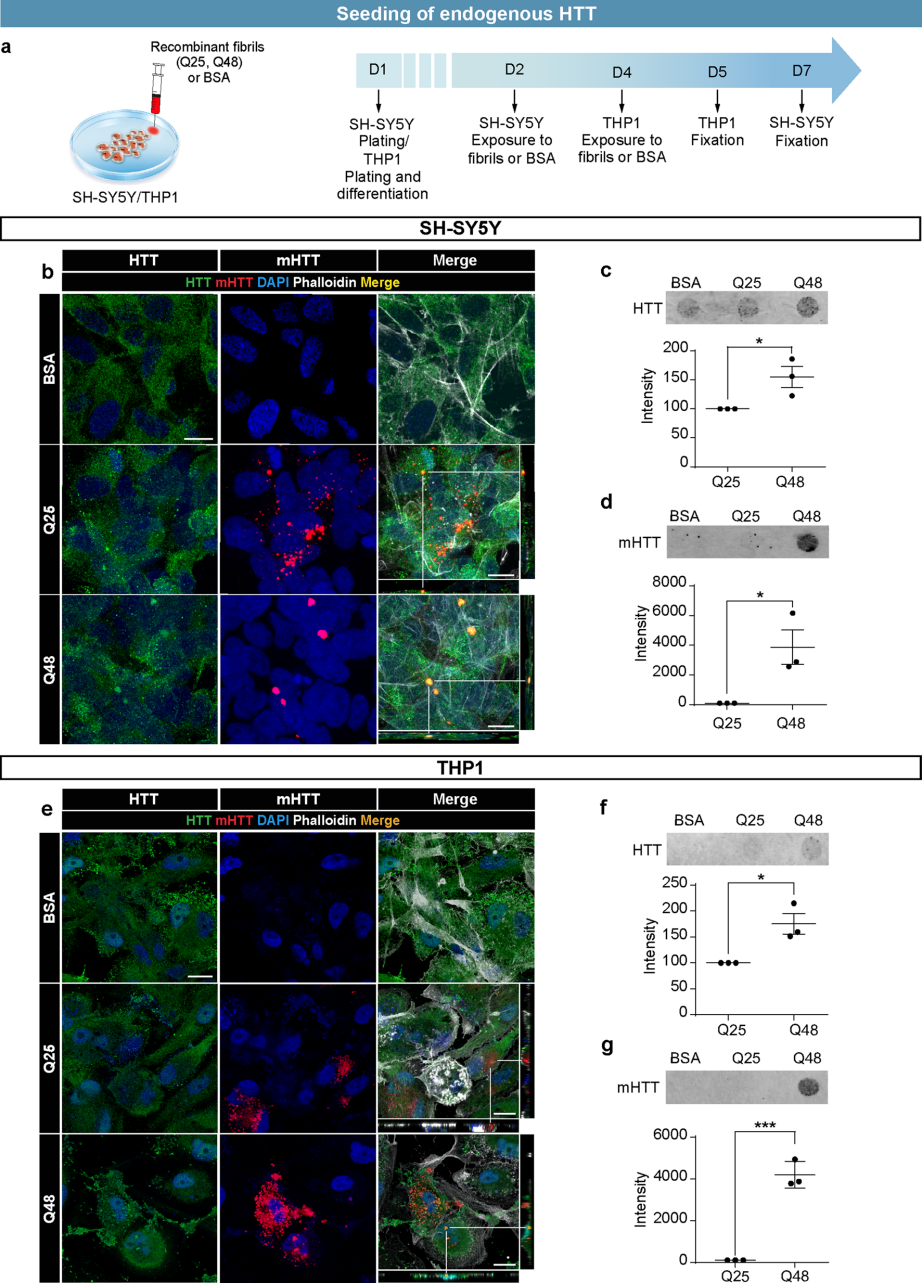
Seeding of endogenous HTT by exogenous HTTExon1Q48 fibrils. Schematic of experimental design for SH-SY5Y cells and THP1-derived macrophages (**a**). Representative confocal photomicrographs of cells exposed to ATTO550-labeled HTTExon1Q25/Q48 fibrils for 5 days (SH-SY5Y—**b**) and 24 h (THP1—**e**). Filter retardation assay and quantification of HTT aggregation immunodetected with anti-WT HTT antibody MAB2166 and anti-aggregated HTT antibody EM48 for SH-SY5Y (**c**, **d**) and THP1 (**f**, **g**) cells. For all filter retardation assay quantifications, the Q48 intensity is shown as percentage of the intensity of Q25. For immunofluorescence, endogenous HTT was detected with MAB2170 (green), HTTExon1Q25 and Q48 (red) and cell nuclei were stained with DAPI (blue). All graphs are the average of three independent experiments. Data are expressed as mean ± SEM. Statistical analysis was performed using a students’ unpaired *t* test (**f**). *p < 0.05, ***p < 0.001. Scale bars = 10 µm. *GFP* green fluorescent protein, *HTT* huntingtin, *BSA* bovine serum albumin

All quantifications of photomicrographs were carried out with FIJI (ImageJ) on confocal images obtained using a 40 X objective from multiple randomly selected regions across the entirety of the coverslip. Quantification of intracellular fibrils or conditioned media-derived mHTT aggregates was performed manually and the position of fibrils/mHTT aggregates was confirmed using the orthogonal projection application of the software. The quantification of fibril size was performed using the “measure particles” function of ImageJ. The total number of cleaved caspase 3 positive cells was obtained using the “cell counter” plug-in of ImageJ. The length of the longest projection emerging from the cell soma (defined as the primary neurite) was measured manually for each cell. The number of cells with secondary projections was counted manually. Filter retardation assay quantifications were carried out with ImageStudioLite software. The intensity of anti-HTT staining after exposure to HTTExon1Q48 is reported as percent change from the corresponding Q25 replicate (Fig. 2).

### Animals

Three distinct animal experiments were conducted, two involving WT mice and one using the R6/2 model of HD. WT male C57BL/6NCrl mice were ordered from Charles River Laboratories at 6 weeks of age and were housed three per cage. WT mice received either intracerebral or intravenous injections. For the intracerebral protocol, mice were maintained for 14 months post-injection with behavioral testing occurring at 1, 2, 3, 6, 10, and 14 months. Behavioral tests performed were the open field, novel object recognition, light–dark box and the ledge test. For intravenously injected mice, behavioral measures were performed at 1, 2 and 3 months. Behavioral tests included the open field, Y-maze and light–dark box (all described below).

R6/2 ovary-transplanted WT females (B6CBA-Tg(HDexon1)62Gpb/3J) were purchased from The Jackson Laboratories. Pups were weaned as usual but underwent intraventricular injection of BSA, HTTExon1 Q25 fibrils or HTTExon1Q48 fibrils at post-natal day (p) 9. Genotyping was performed by polymerase chain reaction (PCR) analysis of DNA obtained from ear samples. Behavioral testing of treated pups began at 1 month and continued until 2 or 3 months of age. Each month, 1–2 mice per group were killed to allow post-mortem analysis at multiple time points. Behavioral tests included the clasping test, cylinder test, grip test, open field, light–dark box, and Y-maze. Two animals per treatment group were killed 1 h post-injection and at the end of each behavioral testing.

All mice were maintained in a temperature and light-controlled environment (22 °C, a 12-h cycle) and all animal experiments were performed in accordance with the Canadian Guide for the Care and Use of Laboratory Animals, and all procedures were approved by the Institutional Animal Care Committee of Université Laval.

#### Intracerebral injections of fibrils

Eight-week-old male C57BL/6NCrl mice (*n* = 28/group) were deeply anesthetized by administration of 5% isoflurane, maintained at surgical plane with a dose of 1.5% isoflurane and kept warm with a heating pad. After the head was firmly fixed to a stereotaxic frame with ear bars, the skin was disinfected by swabbing 70% ethanol and chlorhexidine. A small portion of skin was cut with surgical scissors to expose the skull and permit the identification of bregma. Mice received unilateral injections of 2 µL Q25 or Q48 fibrils (1 µg/µL) into layer V of the motor cortex using the following stereotaxic coordinates: ML: − 1.40, AP: ± 1.60, DV: − 0.75 [40]. Injections were performed using a glass Hamilton syringe (Hamilton company, QC, CA, Cat# FSSP9718951) equipped with a 25-mm-long 31 G needle and a bevel of 30° (Hamilton company, CAT# 7803-03). Following injection, the skin on top of the head was sutured with Vicryl sutures (Ethicon, PR, USA, CAT# J391H), isoflurane was interrupted, and the mice were placed in a recovery cage on a heating pad before being returned to their home cages.

#### Intraventricular injections of fibrils

Male and female WT and R6/2 pups (p9) were prepared as described above. Pups were then injected with 1 µL of solution containing 2 µg/µL of synthetic fibrils Q25 or Q48 or 1 µL of 1 µg/μL BSA bilaterally into the lateral ventricles using the stereotaxic coordinates ML: − 0.90, AP: ± 0.40, DV: − 2.50, as previously published [56]. Injections were performed using a glass Hamilton syringe (Hamilton company, QC, CA, Cat# FSSP9718951) equipped with a 25-mm-long 31 G needle and a bevel of 30° (Hamilton company, CAT# 7803-03). Due to the young age of the pups, the skull surface was soft allowing the passage of the needle through the bone without requiring a microdrill. After the injection, the skin was fixed onto the skull with surgical glue (Vetbond, 3M, USA, Cat# 1469SB), the flux of isoflurane was interrupted, and the mice were placed in a recovery cage before being returned to their mother.

#### Intravenous injections in adult WT mice

Eight-week-old male C57BL/6NCrl mice received intravenous injections of 3.5 µg of Q25 or Q48 fibrils (Q25, *n* = 8; Q48, *n* = 7) in 100 µL of isotonic saline every 2 weeks for 3 months.

### Behavioral assessment

All behavioral tests were performed during the light phase of the light/dark cycle and at the same time of the day throughout the study. The experimenter was blinded for the duration of the protocol. Prior to all behavioral testing, mice were habituated to the testing room for 12 h. The experimenter was present in the room throughout all behavioral tests, with the exception of the open field. All testing arenas were cleaned with 70% ethanol between animals. All behavioral tests were video-recorded and analysis performed off-line from the collected videos with the exception of the open field, which was automatically scored using PAS software and novel object recognition which was scored live.

#### Clasping

The clasping test is a standard measure of HD phenotype [34]. Clasping was analyzed by suspending mice by their tails 10/15 cm above the cage for 30 s. Two different parameters were assessed: (1) the time displaying whole clasping, defined as the retraction of all four limbs toward the abdomen and (2) a score of clasping intensity: 0 = no clasping, 1 = one hind limb retracted toward the abdomen, 2 = both hind limbs retracted toward the abdomen, 3 = all 4 limbs retracted toward the abdomen. We also measured the percentage of animals displaying whole clasping. This test was repeated every week starting from 4 weeks of age.

#### Open field

Mice were tested individually for 60 min in a PAS-home cage system consisting of a square Plexiglas arena (25″ × 25″) equipped with 16 × 16 photo beams that records beam breaks in real time (San Diego Instruments, USA). The distance traveled, average speed, number of rears as well as fine and ambulatory movements in the center of the field and the periphery were retrieved from the PAS software in 5-min bins. These data were used to calculate locomotor activity, anxiety-related behavior as well as short- and long-term memory [8].

#### Cylinder

Mice were placed in a raised transparent glass cylinder (diameter: 11.5 cm; height: 14 cm) for 3 min. Motor behavior was recorded by a camera placed on the bottom of the cylinder to observe how many times the animals touched the ground with the left, the right or both front paws. This was utilized as a test of gross motor function [17].

#### Ledge test

Mice were lifted and placed on the rim at one end of a clean cage. They were filmed throughout the time taken to walk from one end of the ledge to the other, and to descend down into the cage. Scoring was based on their footing: 0—a mouse walked across the ledge with no troubles and lowered itself into the cage, 1—a mouse lost its footing once or twice while walking across the ledge, 2—a mouse was dragging its rear legs as it pulled itself across the ledge, 3—the mouse fell off of the ledge or was unable to lower itself into the cage without falling head-first [23].

#### Grip test

We used an apparatus (Chatillon DFE II Series—Ametek sensor, tests and calibration) consisting of a grid connected to a force meter. Mice were placed such that all four limbs were on the grid allowing the mouse to grip the device. After placement, the experimenter gently pulled the mouse until the animal released its grip on the grid. The force meter automatically registered the force [measured in kilogram-force (kgF)]. This test was used to assess muscle strength [10].

#### Light–dark box

The apparatus consists of two connected compartments of the same dimension (25 × 25 × 25 cm), one black and covered to avoid light, the other open and transparent allowing the passage of light. Mice were placed one at a time in the dark compartment of the box. The time spent in the dark and the time spent in the light were measured as well as the number of head emergences from the door. The test has a total duration of 5 min and was recorded by a camera placed on the side of the apparatus. This test is used to assay the unconditioned anxiety-related behavior as mice are nocturnal animals that prefer darker areas. However, when placed in a novel environment, they have the tendency to explore. The degree of anxiety influences the expression of these two competing drives permitting measurement of anxiety-related behavior [30].

#### Y-maze

The Y-maze apparatus is made of clear acrylic and composed of three equal arms measuring 32.5 cm in length, 8.5 cm in width and 15 cm in height. The walls of the maze are identical and opaque to prevent the utilization of visual spatial clues. Mice were placed one at a time in the middle of the maze and all four limb entries were recorded by a camera for 7 min allowing the experimenter to be out of the mouse’s field of view. A correct alternation was defined as successive entrance into each of the three arms, in any order, without re-entering one of the arms. Percent correct alternation was calculated as the number of alternation divided by the total number of entries minus two [31]. The aim of this test is to evaluate short-term spatial working memory [31].

#### Novel object recognition

Mice were placed in an empty, transparent box measuring 40 cm × 25 cm after habituating to the behavior room for 1 h prior to testing. Two identical glass rectangular prisms were placed at equal distances from the edges of the cage (12 cm). The mouse was placed between the two objects and allowed to explore the cage for 5 min. Two hours later, the experiment was repeated but one of the rectangular prisms was replaced with a glass cylinder with floral decorations. The mouse was once again given 5 min to explore each of the objects. To measure the index of recognition, we examined the time spent observing: Object 2/(Object 1 + 2) [57]; to measure the index of demotivation: Object 1 + Object 2 (2nd observation)/Object 1 + Object 2 (1st observation) [3]. Additionally, the total time the mouse spent observing the objects during both exposures was recorded. Time observation was measured as the amount of time the mouse spent with its head near the object.

### Post-mortem analyses

#### Tissue processing

Mice were killed at 1 h, 4, 8 and 12 weeks post-intraventricular injection, 1, 2, 3 and 14 months post-cortical injection, or 3 months post-intravenous injection. All mice were subjected to intra-cardiac perfusion with PBS followed by perfusion with 4% PFA under deep anesthesia with 1% ketamine (30 mg/kg) and xylazine hydrochloride (4 mg/kg). Brains were collected, post-fixed in 4% PFA overnight and subsequently stored in 20% sucrose in PBS for cryoprotection. For the intra-ventricular injection protocol, the 8 and 12 weeks time points as well as all time points from the other experiments, were cut into  25-μm-thick coronal brain sections using a sliding microtome (Leica Microsystems, ON, CA, Cat# SM 2000R), serially collected in anti-freeze solutions and stored at − 20 °C until use. For intra-ventricular injections, the 1 h and 4 week time points, were cut into 12-μm-thick coronal brain sections using a cryostat (NX 70, Thermo Scientific) and mounted sections were stored at − 20 °C until use.

#### Preparation of plasma free of platelets

During killing, ~ 1 mL of blood was collected by cardiac puncture into tubes containing 200 μL acid citrate dextrose and 350 µL of Tyrode pH 6.5. Tubes were centrifuged for 8 min at 600*g* at RT. Platelet-rich plasma was collected and 1/5 of the volume of acid citrate dextrose and 1/50 of the volume of ethylenediamine tetraacetic acid 0.5 M were added to this, before the complete solution was centrifuged at 400*g* for 2 × 2 min and at 1300*g* for 5 min. The supernatant was then collected and centrifuged at 2500*g* for 15 min to obtain the platelet-free plasma (PFP) for assessment of the antibody titre of animals administered fibrils by intravenous injections.

#### Immunofluorescence for in vivo experiments

Free-floating and mounted sections were washed in PBS (3 × 10 min), incubated in 3% H_2_O_2_ for 30 min, washed (3 × 10 min) and blocked with 10% donkey serum (Sigma-Aldrich), 0.01% Triton X-100 (Sigma-Aldrich) 0.5% BSA (Bioshop) and incubated overnight at 4 °C with anti-HTTExon1 rabbit polyclonal antibody (1:1000, raised in Ronald Melki’s lab using denatured HTTExon1Q45 as the antigen), anti-MAP2 (1:500, LifeSpan Bioscience, WA, USA, Cat# LS-B290-50, or 1:500, Sigma-Aldrich, M1406) and one of the following antibodies; anti-HTT clone EM48 (1:500, Millipore, MAB5374), anti-ubiquitin (1:100, Thermo Fisher Scientific, 13-1600) or anti-HTT (1:1000, Millipore, MAB2170). After primary antibody incubation, samples were washed (3 × 10 min), incubated with secondary antibodies (Alexa Fluor 488, 547, or 647 for the appropriate animal host; 1:500; Thermo Fisher Scientific or Jackson ImmunoResearch, ON, CA, Cat# 703-175-155) diluted in blocking solution for 2 h at RT, washed again, incubated with DAPI nuclear stain (Thermo Fisher Scientific) diluted in PBS to 0.022% at RT for 7 min, washed, mounted using Fluoromount G (Thermo Fisher Scientific) and stored at 4 °C. For the immune-detection involving adult injected animals, mounted sections were kept in 70% Ethanol for 5 min, treated with Autofluorescence Eliminator Reagent (Millipore, Cat# 2160) for 5 min, washed three times in 70% ethanol for 1 min each and then processed, as previously described [39].

#### Enzyme-linked immunosorbent assay

Antibodies against fibrils were tittered in PFP with an indirect homemade *Enzyme*-*Linked Immunosorbent Assay* (ELISA). Seven hundred and fifty nanograms of fibrils in carbonate 50 mM pH 9.6 solution were first coated in a 96-well plate (Sigma-Aldrich, Cat# 3690) for 2 h. The plate was then washed three times with PBS 0.1 M with 0.05% Tween 20 (PBST), blocked with 2% gelatin (Bio Rad, CA, USA, #1706537) in PBS 0.1 M for 2 h at 37 °C and washed three times with PBST. One hundred microlitre of diluted PFP in 0.2% gelatin was incubated overnight at 4 °C (dilution from 10^3^ to 5.10^6^). The plate was then washed four times with PBST and incubated with the secondary antibody conjugated with horseradish peroxidase: goat anti-mouse (1:25,000, Jackson ImmunoResearch, Cat# 115-035-166) diluted in 0.2% gelatine for 2 h at RT. The plate was washed four times with PBST and then 3,3′,5,5′-tetramethylbenzidine (G-Bioscience, Mo, USA, Cat# 00-4201-56) substrate was added for 15 min at RT. The addition of sulfuric acid 0.18 M stopped the reaction and measurement of the intensity at 450 nm was done using a multi-detection microplate reader (Synergy HT; BioTek, VT, USA).

#### Image acquisition and preparation

Fluorescent photomicrographs were obtained using a Zeiss Zen Imaging software linked to a Zeiss Imager Z.2 AXI0 confocal microscope (Zeiss, Oberkochen, Germany). All images were prepared using Adobe Photoshop CS5. When necessary, brightness and contrast adjustments were made. Panels were assembled using Adobe Illustrator CS5.

#### In vivo quantification

All quantifications were performed on pre-defined regions (prefrontal cortex, cerebral cortex, striatum, hypothalamus and hippocampus) across the entire brain. For the quantification of endogenous HTT by immunofluorescence, WT adult mice killed 14 months post-surgery and R6/2 and WT littermates killed at 12 weeks of age, were assessed. An entire series of sections encompassing the entire rostral caudal area of the brain was mounted on a slide and every third section of the slide was imaged with the distance between each section corresponding to a total distance of ~ 750 µm. In this quantification, we analyzed the area stained between different brain regions and treatment groups. Immunostaining was completed in two batches. To correct for differences between batches, data are shown as percent of Q25-treated WT mice. To further assess changes to endogenous HTT, the number of HTT aggregates was counted. These events were rare and we, therefore, analyzed the aforementioned regions of the brain through the binocular lens of the confocal microscope using the 20 × objective, without sampling or acquiring pictures. Endogenous HTT is visible as a diffuse protein within all the cellular compartments. We considered any agglomeration of signal to be an aggregate. Finally, we detected EM48 and the HTTExon-1 in R6/2 mice injected with BSA, Q25 and Q48 fibrils by immunofluorescence at 12 weeks of age. For this quantification, the colocalization between HTTExon-1 and EM48 inclusions was quantified for Q25- and Q48-injected mice using the cell count plug-in in FIJI (ImageJ) photomicrographs. Colocalization was defined by overlap of different emission channels in multiple focal planes through each brain section.

### Statistical analysis

For in vitro experiments, all statistics were performed using a students’ unpaired *t* test. For the in vivo experiments, two-way ANOVAs followed by Tukey’s post hoc tests were used for analyses of R6/2 and WT mice at a single time point, while one-way ANOVAs followed by Tukey’s post hoc tests were used when comparing three treatment groups of one genotype at single time point. Analysis of a single genotype across multiple time points was performed using a repeated measures two-way ANOVA, excluding animals that were not tested at all time points. A linear mixed-effects model was implemented to assess change overtime for two genotypes as a function of treatment group and genotype. Within-subjects variance was controlled for by including random effects of intercept and slope for each mouse. The model was estimated using maximum likelihood and contrast comparisons were performed to determine the effect of treatment and genotype at each time point. Analyses were performed using RStudio version 3.4.1 with nlme version 3.1-131. All data are expressed as mean ± SEM. For all experiments, a significance cut-off of 0.05 was used. Graphs and statistical analysis were performed using the Prism software (v6.01; GraphPad Software, San Diego, CA) unless otherwise specified. For all *t* tests and one-way ANOVA’s, equal variance was confirmed by Fisher’s test and where variances were unequal, a Mann–Whitney test or Kruskal–Wallis test was used.

## Results

### mHTTExon1 fibrils of human origin are toxic to multiple cells lines

To explore the spreading and toxicity of mHTTExon1, we opted to use recombinant HTT/mHTT fibrils which correspond to the product of exon 1 of the HTT gene. Fibrillar mHTT has previously been shown to be particularly toxic and prone to aggregate formation [42]. This makes this form an excellent candidate for understanding the potential of mHTT to spread and seed as it has the highest propensity to form aggregates. Prior to use, fibrils were imaged by electron microscopy. Both non-pathogenic Q25 (Online Resource 1a) and pathogenic Q48 fibrils (Online Resource 1b) were present in the solution and displayed the appropriate fibrillar structure [38]. After confirming the presence of fibrils, a dose response curve was performed in a human neuroblastoma-derived cell line (SH-SY5Y) and 0.005 µg/μL was selected as the working concentration (Online Resource 1c). This concentration (0.005 µg/μL) fell in the center of the curve where minimal toxicity was observed. This intermediate concentration allowed us to limit effects on cell survival in long-term exposure experiments, while still maintaining a sufficient concentration to induce a cellular effect. SH-SY5Y cells were then incubated with HTTExon1Q25-ATTO488, HTTExon1Q48-ATTO488 fibrils, or BSA-ATTO488 for 72 h (Fig. 1a). After exposure to fibrils, cells were fixed, immunostained for cleaved caspase 3 as well as phalloidin to visualize the cell membrane. Using confocal microscopy, the presence of both Q25 and Q48 fibrils was identified within cells (Fig. 1b). In agreement with previous findings by Ren et al. [45], quantification of the percentage of cells containing fluorescent puncta indicated that, while Q25 and Q48 fibrils were present within cells, there was a far greater number of cells containing Q48 fibrils when compared to Q25 fibrils (Fig. 1c). Furthermore, cells exposed to Q48 fibrils had significantly more puncta per cell than did cells exposed to Q25 (Fig. 1d). The size of the puncta within the cells did not, however, differ between the two conditions (Online Resource 1e). Fibrils had a toxic effect in both Q25 and Q48 conditions, but Q48 was significantly more toxic than Q25 fibrils (Fig. 1e). This increased toxicity was unlikely to be secondary to the increased number of cells containing Q48 fibrils, as little to no cleaved caspase 3 activity was detected in SH-SY5Y cells containing Q25 fibrils. Contrary to this, 1% of Q48 fibril containing cells were undergoing apoptotic cell death (Online Resource 1f).

While neurons are among the most affected cells in HD, other populations, including microglia [13], have been implicated in pathology. To determine if fibrillar mHTTExon1 also impacted different cellular populations, we exposed human THP1-derived macrophages to Q25 and Q48 fibrils for 24 h (Fig. 1a, f). Similarly to what was observed in SH-SY5Y cells, THP1-derived macrophages incorporated fibrils from the media (Fig. 1f). More cells contained Q48 than Q25 fluorescent puncta (Fig. 1g) and more puncta per cell were present in cells exposed to Q48 than Q25 fibrils (Fig. 1i). Macrophages did, however, differ from the neurons in few important aspects. The number of puncta per cell was higher in macrophages than in SH-SY5Y cells (Fig. 1d, h), the size of the puncta was larger in cells exposed to Q48 as compared to Q25 fibrils (Online Resource 1g), and there was no overall difference in the total number of apoptotic cells between Q25- and Q48-treated macrophages (Fig. 1i). Importantly, there was still a difference in the toxicity of Q25 and Q48 fibrils, but this was only detectable in cells containing fibrils. In this population, there were also a higher percentage of Q48-containing cells undergoing apoptosis than Q25-containing cells (Online Resource 1h).

The similar findings we report in two distinct cell lines and previous publications [45] corroborates that Q25 and Q48 fibrils can be taken up and that they both can exert an effect on cells. However, there are a number of differences between cell lines and primary cultures. To confirm our findings in a more physiological relevant condition, iGABA human neurons derived from induced pluripotent stem cells were utilized. The iGABA cells were exposed to HTTExon1 fibrils or BSA for 3 days (Online Resource 1d). Similar to the cell line results, we observed uptake of fibrils from the cellular media (Online Resource 1i). Unlike the two cell lines, the number of iGABA neurons containing Q25 fibrils did not differ from the number of neurons containing Q48 fibrils (Online Resource 1j). Despite the similarity in the number of cells containing the two types of fibrils, there were still differences in cellular effects as cells exposed to Q48 fibrils were characterized by a significant decrease in the length of primary neurites (Online Resource 1k) and fewer cells depicted secondary neurites than cells exposed to Q25 fibrils (Online Resource 1l). These results confirm that fibrils can be uptaken from media into human cell lines in a number of different conditions.

### Fibrils can recruit WT HTT into aggregates

To evaluate the prion-like capacity of human recombinant HTTExon1 fibrils, we assessed the state of endogenous HTT in SH-SY5Y cells. SH-SY5Y cells were treated with fibrils for 5 days (Fig. 2a) and subsequently either fixed or homogenized. Fixed cells were immunostained for endogenous WT HTT to visualize changes in protein conformation (Fig. 2b). To better facilitate quantification, the presence of aggregated HTT was assessed in cell lysates using a filter retardation assay. Increased aggregation of HTT was observed in cells exposed to Q48 compared to Q25 or BSA (Fig. 2c, d).

To determine if this process was specific to neuronal-like cells, THP1 differentiated macrophages were exposed to fibrils for 24 h prior to fixation and immunofluorescence detection of endogenous HTT (Fig. 2d). This shorter treatment duration was selected for macrophages as they demonstrated faster uptake kinetics. After 24 h of treatment with Q48 fibrils, changes in the staining pattern of WT HTT were already clearly visible (Fig. 2e). While colocalization and aggregation were apparent in the THP1 cells, we selected to quantify aggregation using a filter retardation assay. Similar to the data obtained for SH-SY5Y cells, we observed an increase in the intensity of WT HTT levels (Fig. 2f) and mHTT aggregates (Fig. 2g) after exposure to Q48 fibrils (Fig. 2f, g). Together, these two cell lines provide strong support for prion-like effects of Q48 fibrils in vitro.

### Intracerebral injection of HTTExon1Q48 fibrils induces cognitive deficits and anxiety-like behavior in WT mice

**Fig. 3:**
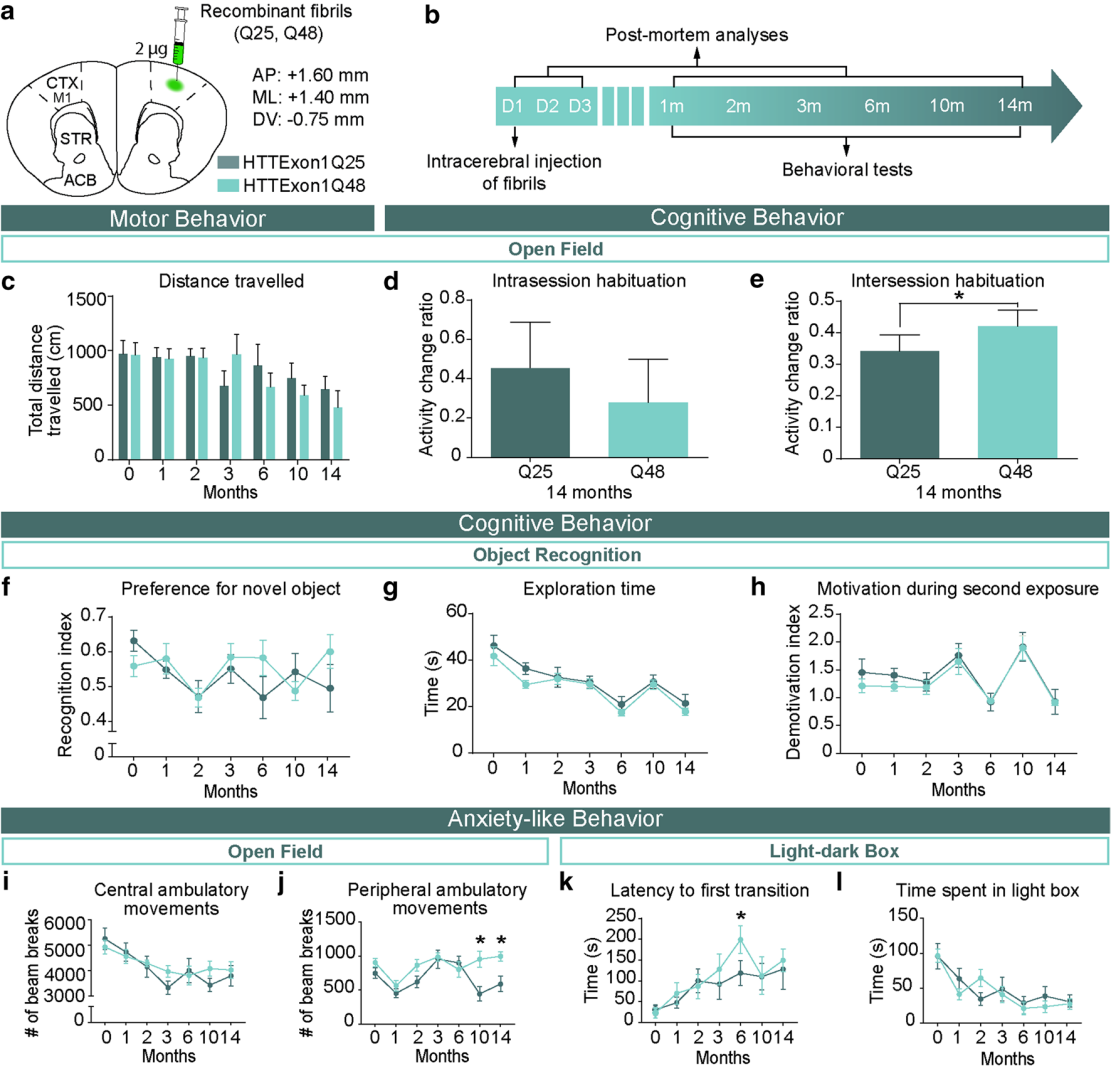
Manifestation of behavioral impairments in adult WT mice injected with HTTExon1Q48 fibrils. Atlas coordinates for intracerebral injections, treatment legend (**a**) and experimental timeline (**b**). Animals underwent motor, cognitive and anxiety-like behavioral tests from 1 to 14 months post-injection. Motor behavior was assessed using total distance traveled in 60 min in the open field (**c**). At 14 months post-injection, short-term memory was evaluated by assessing the change in distance traveled between the first 5 and the last 5 min of testing (**d**) and long-term memory was assessed by calculating the change in distance traveled in the first 5 min of baseline to 14 months post-injection (**e**). Cognitive performance was assessed at each time point by measuring the preference for the novel object (**f**). The absence of confounding factors was determined by measuring the exploration time (**g**) and motivation during the exposure to the novel object (**h**). Anxiety-like behavior was assessed in the open field by quantifying the distance traveled in the center of the open field (**i**) and in the periphery (**j**) as well as in the light–dark box by latency to leave the dark box (**k**) and total time spent in the light box (**l**). Data are expressed as mean ± SEM. Baseline HTTExon1Q25 *n* = 12, HTTExon1Q48 *n* = 12; 1 month HTTExon1Q25 *n* = 11, HTTExon1Q48 *n* = 11; 2 months HTTExon1Q25 *n* = 10, HTTExon1Q48 *n* = 10; 3–14 months HTTExon1Q25 *n* = 9, HTTExon1Q48 *n* = 9. Statistical analysis was performed using a students’ unpaired *t* test for individual time points and a two-way ANOVA followed by Tukey’s post hoc test for across time graphs. *p < 0.05. *ACB* nucleus accumbens, *AP* antero-posterior, *CTX M1* primary motor cortex, *D* day, *DV* dorso-ventral, *m* month, *ML* medio-lateral, *STR* striatum

To determine if prion-like properties of the abnormal HTT protein extended to in vivo conditions, WT mice received intracerebral injections of either HTTExon1Q25 or Q48 fibrils at 2 months of age (Fig. 3a). Injected mice underwent a battery of behavioral tests at multiple time points (1, 2, 3, 6, 10, and 14 months) extending out to 14 months post-surgery (Fig. 3b). The test battery included measures of motor function, cognition, and anxiety-like behavior as all of these behaviors are known to be altered in HD patients [50]. To assess motor performance, mice were tested in both the open field and ledge tests. No significant differences were detected between groups on either test at any time point (Fig. 3c and Online Resource 2a). Mice were also tested for cognitive deficits using the open field and novel object recognition. In the open field, cognition was subdivided into short- and long-term memory by assessing both habituation during one 60-min trial (intrasession) and between trials (intersession habituation) in the open field, respectively. No difference in short-term memory was observed 14 months post-surgery (Fig. 3d). However, Q48-treated mice did demonstrate statistically significant impaired long-term memory at 10 (data not shown) and 14 months post-surgery (Fig. 3e). Unlike the open field data, no difference in long-term memory was observed when measured using preference for the novel object (Fig. 3f). Exploration time (Fig. 3g) and motivation during the novel object testing phase (Fig. 3h) did not differ between groups, which indicates that no motor confounds or differences in motivation influenced preference for the novel object. Finally, mice were also assessed for anxiety-like behavior using the open field and the light–dark box. In the open field, no change was detected in central ambulatory movements (Fig. 3i); however, a mild increase in distance traveled in the periphery was observed 10 months post-surgery (interaction: *F*_6,118_ = 2.263, *p *< 0.05) (Fig. 3j). The tendency to remain close to the walls is known as thigmotaxis and is indicative of anxiety-like behavior in mice [30]. The presence of an anxiety-like phenotype was further supported by an increased latency to leave the dark compartment at 6 months post-surgery (time: *F*_6,120_ = 5.176, *p* < 0.001,Treatment: *F*_1,120_ = 1.628, *p* > 0.05) (Fig. 3k). The anxiety phenotype was mild as it did not extend to a change in time spent in the light compartment (Fig. 3l), exploration (Online Resource 2b), rearing (Online Resource 2c), or latency to first emergence of the head (Online Resource 2d). The presence of cognitive impairment and anxiety-like behavior in Q48-injected mice supports the in vitro data showing toxicity and prion-like capacity of exogenously administered Q48 HTT fibrils.

### Changes in the staining patterns of endogenous HTT in adult WT mice

**Fig. 4:**
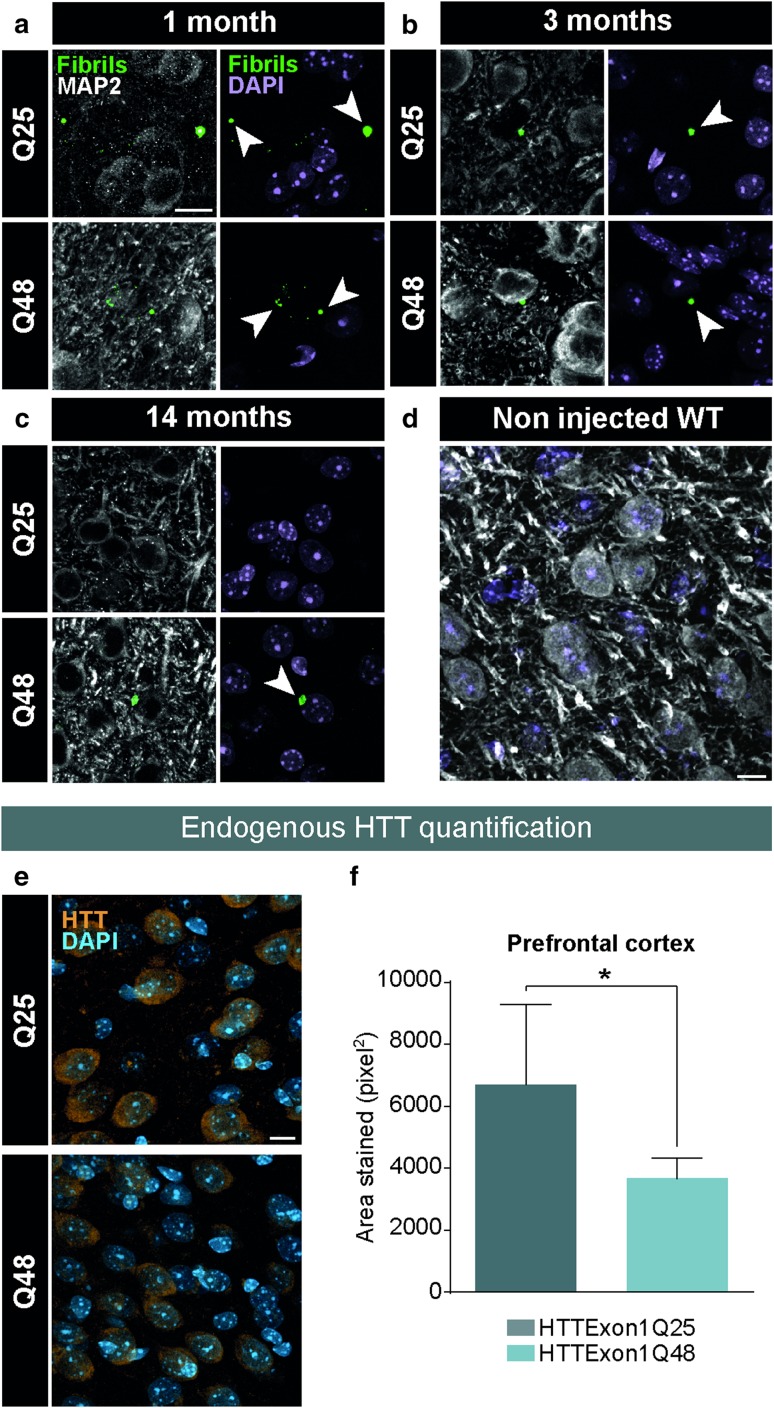
Post-mortem identification of HTTExon1Q25 and Q48 fibrils in adult WT mice. Representative confocal photomicrographs of brain tissue of WT mice sacrificed 1 month (HTTExon1Q25 *n* = 2, HTTExon1Q48 *n* = 2) (**a**), 3 months (HTTExon1Q25 *n* = 2, HTTExon1Q48 *n* = 2) (**b**) and 14 months (HTTExon1Q25 *n* = 8, HTTExon1Q48 *n* = 8) post-injection and immunostained for HTTExon1 (green) and MAP2 (white). Arrowheads indicate the localization of fibrils. Specificity of anti-HTTExon1 antibody was assessed by staining a non injected WT mouse (**d**). Representative confocal photomicrographs of brain tissue of WT sacrificed at 14 months post-surgery and immunostained for endogenous HTT (orange) (**e**). Quantification of HTT intensity as measured by total area stained in the prefrontal cortex (**f**). Nuclei were detected with DAPI (purple **a**–**d**; blue **e**). Data are expressed as mean ± SEM. WT Q25 *n* = 7, WT Q48 *n* = 7. Statistical analysis was performed using a students’ unpaired *t* test. *p < 0.05. Scale bars = 10 µm. *MAP2* microtubule-associated protein 2, *WT* wild type. Arrowheads indicate the localization of fibrils

To determine if the changes in behavior are due to human HTTExon1Q48 fibrils or their seeding propensity, post-mortem analyses were performed at different time points post-injection to track the localization of fibrils and to determine if changes in endogenous HTT were present. At early time points (1 month), fibrils were readily detectable using a fibril-specific antibody [38] (Fig. 4a). At later time points (3 months), fibrils became progressively more difficult to identify (Fig. 4b) and were no longer detectable for HTTExon1Q25 by 14 months post-surgery (Fig. 4c). To ensure that the staining observed was specific to the fibrils and to exclude the possibility of cross-reactivity with endogenous HTT, non injected WT mice were also immunostained with the anti-exon 1 antibody and no punctate staining was present (Fig. 4d). Given that the fibrils are mostly absent from the brain at the time points where behavioral changes were detected, it was particularly important to understand if HTTExon1Q48 fibrils were capable of inducing changes in the staining pattern of endogenous HTT. Interestingly, a change in the amount of positive HTT signal was detected between groups, with HTTExon1Q48-injected mice having reduced signal compared to their Q25-injected counterparts 14 months post-surgery (Fig. 4e). These changes reached statistical significance in the prefrontal cortex (Fig. 4f), although the more posterior cortical regions also showed a trend towards a decrease (data not shown). The changes in behavior and endogenous HTT staining patterns, even after the clearance of the fibrils, are consistent with mHTTExon1 fibrils possessing prion-like behavior.

### Injection of HTTExon1Q48 fibrils precipitates disease in the R6/2 mouse model of HD

**Fig. 5:**
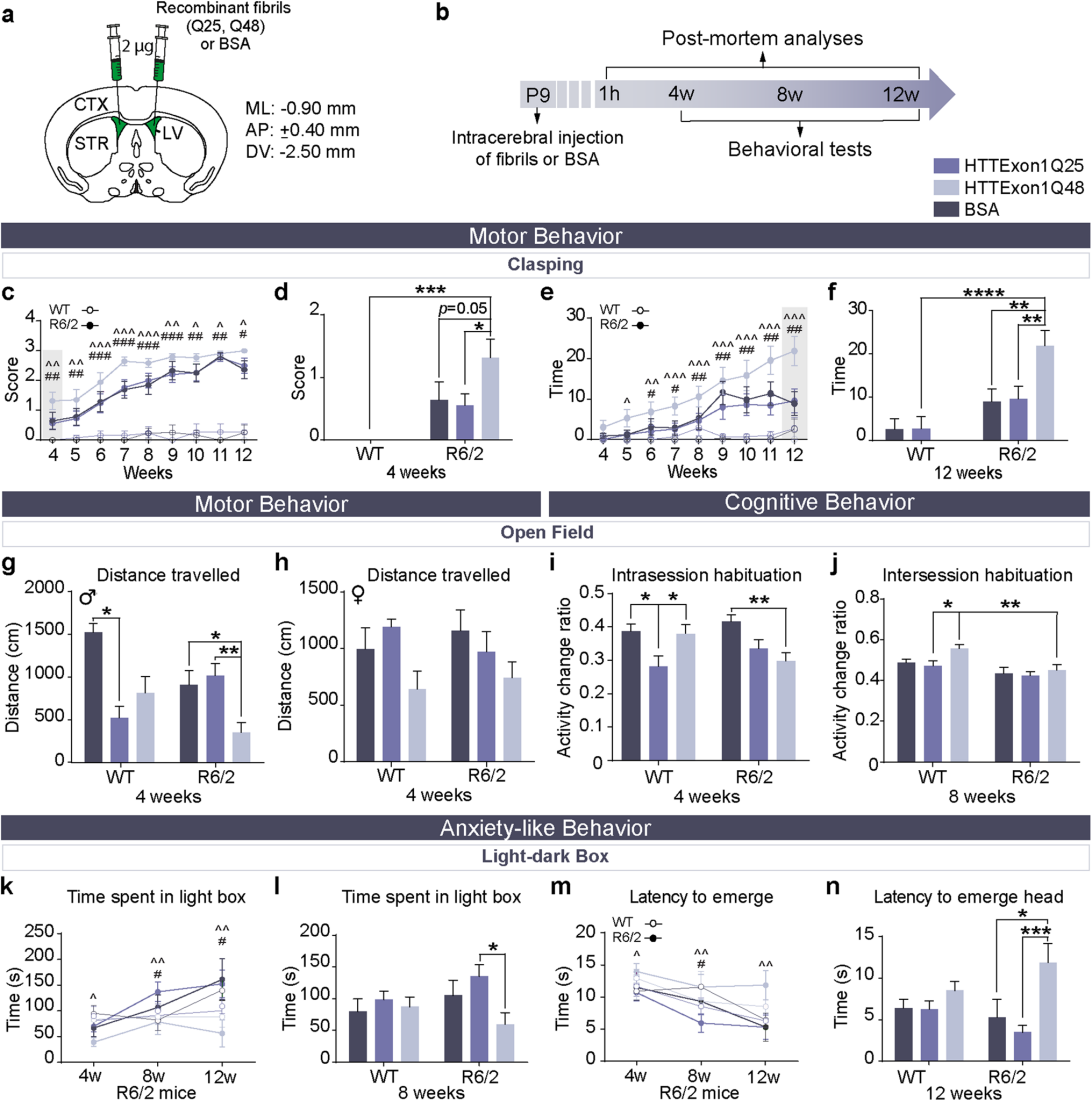
Precipitation of behavioral phenotype in R6/2 mice following injection of HTTExon1Q48 fibrils. Atlas coordinates for intraventricular injections, treatment legend (**a**) and experimental timeline (**b**). Clasping score in R6/2 mice at all tested time points (**c**) and at 4 weeks of age (**d**). Duration of full-clasping behavior at all tested time points (**e**) and at 12 weeks of age (**f**) WT BSA *n* = 13–14; WT HTTExon1Q25 *n* = 11–13; WT HTTExon1Q48 *n* = 12–15; R6/2 BSA *n* = 11–14; R6/2 HTTExon1Q25 *n* = 12–18; R6/2 HTTExon1Q48 *n* = 9–19. Motor endurance was measured by assessing the distance traveled in the last 5 min of the open field at 4 weeks post-injection for male (**g**) and female mice (**h**). WT BSA *n* = 9F, 4M; WT HTTExon1Q25 *n* = 3F, 10M; WT HTTExon1Q48 *n* = 8F, 9M; R6/2 BSA *n* = 7F, 9M; R6/2 HTTExon1Q25 *n* = 7F, 10M; R6/2 HTTExon1Q48 *n* = 11F, 8M. At 4 weeks post-injection, short-term memory was evaluated by assessing the change in distance traveled between the first 5 min and min 25–30 of testing (**i**) and at 8 weeks post-injection, long-term memory was measured by calculating the change in distance traveled between the first 10 min of testing at 4 and 8 weeks post-injection (**j**). WT BSA *n* = 12–13; WT HTTExon1Q25 *n* = 12–13; WT HTTExon1Q48 *n* = 14–15; R6/2 BSA *n* = 12–16 R6/2 HTTExon1Q25 *n* = 16–17; R6/2Q HTTExon148 *n* = 16–19. Anxiety-like behavior was assessed in the light–dark box using time spent in the light box at 4, 8 and 12 weeks of age (**k**) and at 8 weeks only (**l**) and latency to emerge head at 4, 8 and 12 weeks of age (**m**) and at 12 weeks only (**n**). WT HTTExon1Q25 *n* = 11–14; WT HTTExon1Q48 *n* = 11–17; R6/2 HTTExon1Q25 *n* = 8–14; R6/2 HTTExon1Q48 *n* = 8–17. Data are expressed as mean ± SEM. For **c**, **i**, **k**, and **m**, statistics were calculated using a linear mixed effects model. For all other graphs, statistics were performed using a two-way ANOVA with Tukey’s post hoc test. *p < 0.05, **p < 0.01. *AP* antero-posterior, *CTX* cortex, *DV* dorso-ventral, *LV* lateral ventricle, *ML* medio-lateral, *STR* striatum, *w* weeks, *WT* wild type, *M* male, *F* female

Although experiments conducted in adult WT mice show that exogenous HTTExon1 fibrils have seeding propensity, the behavioral impairments observed were subtle and took several months to manifest. In a second set of experiments, mHTT fibrils or BSA, as a non-toxic protein control, were injected bilaterally into the lateral ventricle of R6/2 and WT pups 9 days after birth (Fig. 5a). The R6/2 model of HD has a very rapid disease course [34]; therefore, mice were followed for much less time (12 weeks) (Fig. 5b) than the aforementioned adult WT animals (14 months). HD mice underwent a battery of motor and non-motor tests, similar to what was performed for WT animals. Differences in performance between R6/2 mice injected HTTExon1Q25, HTTExon1Q48 or BSA were detected as early as 4 weeks of age on the clasping test (Fig. 5c, d). At this age, mice injected with HTTExon1Q48 fibrils had an increased average clasping score which was maintained until 12 weeks of age. At later time points, this difference was less striking as the prevalence of clasping behavior increased in Q25 and BSA-injected controls as the disease progressed (Fig. 5c). While the difference between groups diminished over time for clasping scores, the difference between groups for time spent clasping increased (Fig. 5e), with HTTExon1Q48-injected mice displaying a highly statistically significant difference at 12 weeks of age (Fig. 5f). Increased motor impairment was not present in the cylinder test (Online Resource 3a) or grip test (Online Resource 3b), although HTTExon1Q48-injected mice did display a slight increase in the number of wall contacts at 8 weeks of age (Online Resource 3a). This phenotype was transitory and no difference between any groups was observed at 4 or 12 weeks of age. A subtle motor phenotype was, however, observed in the open field at 4 weeks of age. Male R6/2 mice that were injected with HTTExon1Q48 moved less in the last 5 min of testing, which is likely due to increased motor fatigue in the later time points in this 60-min test (Interaction: *F*_2,42_ = 5.10, *p* < 0.05) (Fig. 5g). No such difference was observed in female mice (Fig. 5h). In contrast to the results in adult WT mice, no changes in intersession or intrasession habituation were observed between R6/2 mice injected with HTTExon1Q48 fibrils and with HTTExon1Q25 fibrils (Fig. 5i, j). However, there was a difference in cognitive performance between WT littermates injected with HTTExon1Q48 and Q25 fibrils in short-term memory at the 4-week time point (interaction: *F*_2,85_ = 4.117, *p* < 0.05) (Fig. 5i) and long-term memory at the 8-week time point (genotype: *F*_1,74_ = 12.83, *p* < 0.001, treatment: *F*_2,74_ = 3.152 *p* < 0.05) (Fig. 5j). In both the WT and R6/2 groups, BSA-treated mice demonstrated a transient cognitive impairment at 4 weeks (Fig. 5i) which was lost by 8 weeks of age (Fig. 5j). This transient impairment may be the result of an exaggerated immune response to the presence of BSA in the brain, as previously reported [25]. We additionally observed a lower activity change ratio in Q48-injected R6/2 mice at the 8-week time point. Generally, this is indicative of improved memory but the extreme motor phenotype of R6/2 mice suggests that this decrease could instead be caused by motor fatigue in these animals. In a second well-validated cognitive test, the Y-maze, no change in working memory was observed at the 4-week time point where the decrease in distance traveled was evident. At later time points, R6/2 mice injected with HTTExon1Q48 had a reduced percentage of correct entries as compared to BSA-injected R6/2 mice and Q48-injected WT littermates (genotype: *F*_1,55_ = 13.54, *p* < 0.001, treatment: *F*_2,55_ = 3.553 *p* < 0.05) (Online Resource 3c). Q25-injected R6/2 mice did not significantly differ from either BSA or Q48-injected R6/2 mice. These mice appeared to be half-way between the two treatment groups, suggesting that Q25 may slightly impair cognition at this time point, but that Q48 has a more severe effect. This was the only test where Q25 appeared to exacerbate disease in R6/2 mice.

Anxiety-like behavior demonstrated premature manifestation in R6/2 mice injected with HTTExon1Q48 fibrils with these mice displaying increased anxiety-like behavior as compared to HTTExon1Q25 and BSA-injected mice which displayed decreased anxiety-like behavior at 8 and 12 weeks (Fig. 5k). The performance of Q25- and BSA-injected mice is consistent with the progression of anxiety-like behavior reported in previous studies [7]. At 8 weeks of age, the difference between time spent in the light box by HTTExon1Q25 and HTTExon1Q48 was significant as assessed by two-way ANOVA (Fig. 5l). If all time points were considered in the analysis, both BSA and Q25 significantly differed from Q48-injected R6/2 mice. Anxiety-like performance was additionally measured using a second parameter, namely the time at which the head of the mouse enters the light box. This measure was consistent with the time spent in the light box with HTTExon1Q25, relative to HTTExon1Q48, showing a significant reduction latency to emerge at all time points (Fig. 5m). This difference was preserved up to 12 weeks of age (end of the experiment) where BSA- and HTTExon1Q25-injected mice both displayed a decreased latency as compared to Q48-injected mice (Fig. 5n).

### Exogenous HTTExon1Q48 fibrils colocalize with endogenous mHTT in R6/2 mice

The rapid disease course of R6/2 mice obliged us to conduct the post-mortem analysis 3 months post-surgery. However, this earlier time point increased the detectability of fibrils within animal brains, as the majority of fibrils were not cleared from the central nervous system by this time point. Furthermore, the presence of N-terminal mHTT likely increases the seeding propensity of exogenous HTTExon1 fibrils. This was assessed by immunodetection of endogenous mHTT with the EM48 antibody and fibrils with the anti-HTTExon1 antibody. Double immunofluorescence measurements demonstrated lack of colocalization of exogenous HTTExon1 fibrils and endogenous HTT in WT (Online Resource 4a). In contrast, colocalization was evident in R6/2 mice treated with HTTexon1Q25 or Q48 fibrils by 4 weeks of age (Online Resource 4b). Colocalization cannot be an artifact resulting from poor antibody specificity as it is only observed in R6/2 mice that were treated with fibrils. We nonetheless performed a number of antibody controls. First, to confirm that EM48 does not detect WT HTT, non injected WT mice were immunostained with EM48 and no signal was detected. Similarly, the specificity of the antibody used to detect fibrils was confirmed by immunostaining in non injected R6/2 mice. This control further demonstrated the absence of positive signal (Online Resource 5a). The absence of colocalization in Q25- and Q48-injected WT mice suggests that there was no cross-reactivity between fibrils and endogenous mHTT. We further used ubiquitin as a second antibody to detect endogenous mHTT aggregates in R6/2 mice, as previously described [6]. The same non injected controls performed for EM48 were also stained for ubiquitin (Online Resource 5b) and, they again, confirmed antibody specificity. WT and R6/2 mice were further incubated with anti-ubiquitin. The absence of colocalization between ubiquitin staining and fibrils in Q48-treated WT mice and the presence of colocalization in Q48-treated R6/2 mice further validated the results obtained with EM48 (Online Resource 5c).

**Fig. 6:**
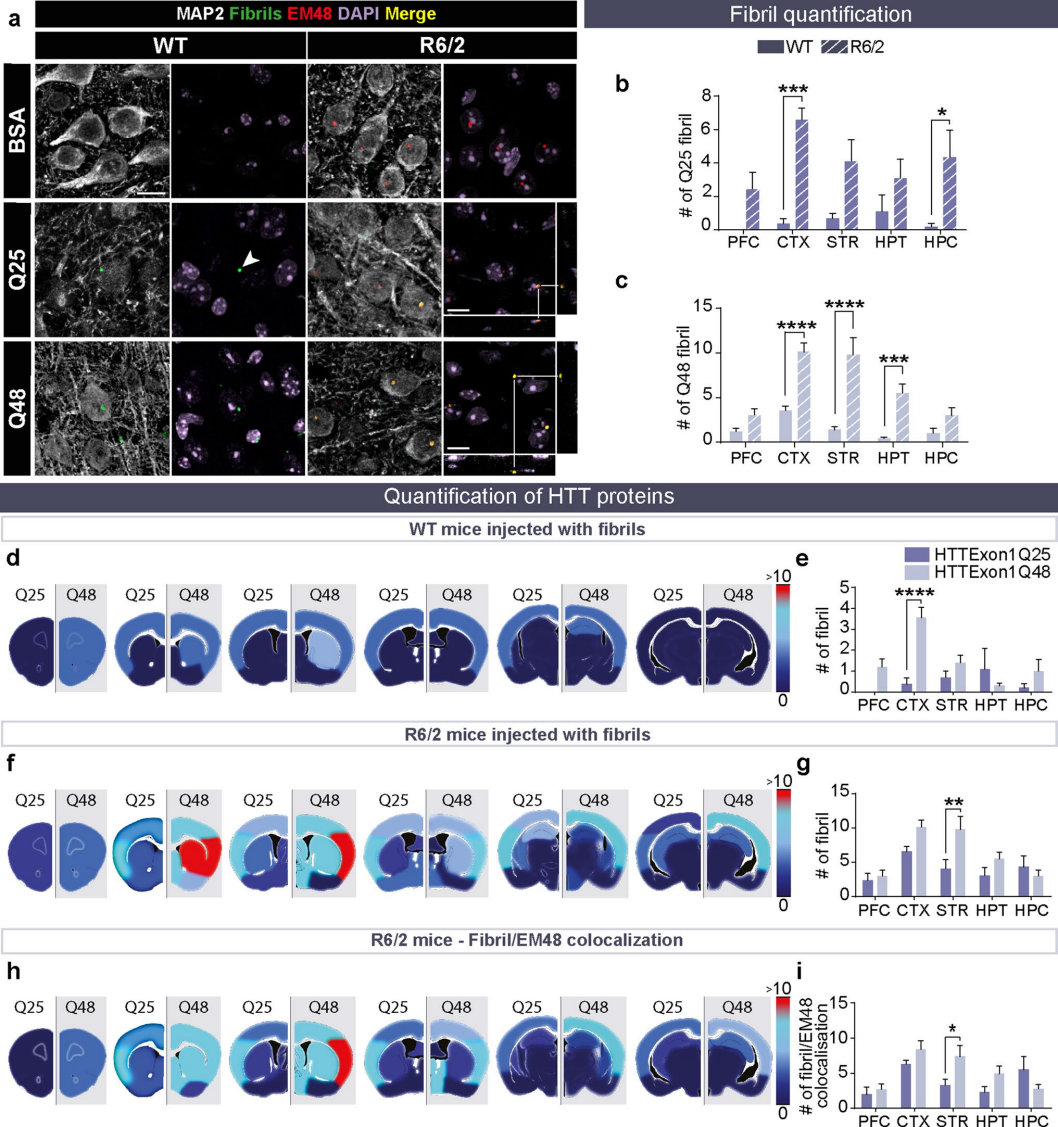
Colocalization of HTTExon1Q48 fibrils and endogenous mHTT in R6/2 mice. Representative confocal photomicrographs of fibrils in the brains of WT and R6/2 mice at 12 weeks post-injection (**a**). Arrowheads indicate the localization of fibrils. BSA-injected mice were used as negative controls to set the lasers on the confocal microscope. Direct comparison of puncta number between WT and R6/2 mice injected HTTExon1Q25 (**b**) and HTTExon1Q48 (**c**). Heat maps depicting the number of puncta detected in different brain regions by converting low numbers of puncta to dark blue, and high numbers to red, in WT (**d**) and R6/2 mice (**f**). The corresponding graphs are displayed for WT (**e**) and R6/2 mice (**g**). The colocalization between EM48 and HTT fibrils depicted as a heat map (**h**) and graph (**i**). Quadruple immunofluorescence of injected fibrils (green), endogenous aggregates EM48 (red), microtubule-associated protein MAP2 (white) and cell nuclei DAPI (purple). Scale bars = 10 µm. Data are expressed as mean ± SEM. WT HTTExon1Q25 *n* = 5, WT HTTExon1Q48 *n* = 5, R6/2 HTTExon1Q25 *n* = 5, R6/2 HTTExon1Q48 *n* = 5. Statistics are performed using a two-way ANOVA with Tukey’s post hoc tests. *p < 0.05, **p < 0.01, ***p < 0.001, ****p < 0.0001. *BSA* bovine serum albumin, *CTX* cortex, *HPC* hippocampus, *HPT* hypothalamus, *MAP2* microtubule associated protein, *PFC* prefrontal cortex, *STR* striatum, *WT* wild type

At the endpoint of this experiment, fibrillar puncta were readily detected in all fibril-treated groups (Fig. 6a). R6/2 mice had more fibrils remaining in the brain 12 weeks after injection as compared to WT mice (Fig. 6b, c). The cortex and hippocampus displayed the most striking differences, although an overall effect of genotype was detected in mice (WT and R6/2) exposed to HTTExon1Q25 fibrils (genotype: *F*_1,40_ = 39.93, *p* < 0.0001) (Fig. 6b). Whereas the cortex, striatum and hypothalamus exhibited the greatest difference (interaction: *F*_4,40_ = 5.185, *p* < 0.01) in WT and R6/2 mice injected with HTTExon1Q48 fibrils (Fig. 6c), the localization of mHTT puncta also differed. In WT mice, most fibrils were detected outside of cells, while in R6/2 mice, fibrils were most often identified within MAP2^+^ cells. This was not the case for HTTExon1Q25 fibrillar puncta, which were equally found inside and outside the MAP2^+^ cells in WT and R6/2 mice (Online Resource 6a). Astrocytes and microglia were also assessed for the presence of fibrils but none were detected in either cell type. When Q25 fibrils were compared with Q48 fibrils within one genotype, puncta were less frequently seen in WT mice that were injected with HTTExon1Q25 than those that received the Q48 fibrillar counterpart (Fig. 6d), suggesting that exogenous HTTExon1Q25 is processed (e.g., cleared or degraded) to a much higher extent than HTTExon1Q48 fibrils in the cortex (interaction: *F*_4,40_ = 5.106, *p* < 0.01) (Fig. 6e). A similar pattern of clearance was observed in R6/2 mice (Fig. 6f). Interestingly, the number of exogenous HTTExon1Q48 puncta was significantly higher than that of HTTExon1Q25 puncta in the striatum of R6/2 animals (fibrils: *F*_1,40_ = 8.359, *p* < 0.01; region: *F*_4,40_ = 7.877, *p* < 0.0001) (Fig. 6g). The seeding capacity of exogenous HTTExon1 was subsequently evaluated by quantifying exogenous and endogenous mHTT aggregates colocalization in R6/2 mice. Regions with the highest number of exogenous puncta tend to display more aggregated (interaction: *F*_4,39_ = 2.727, *p* < 0.05) (Fig. 6h). This was particularly evident in the striatum where a significantly higher colocalization was observed in mice injected with HTTExon1Q48 compared to HTTExon1Q25 fibrils (Fig. 6i).

While increased colocalization between exogenous fibrillar and endogenous mHTT indicates that two pathological proteins may interact, true seeding requires interaction between a WT soluble protein and mutant seeds. Consequently, we assessed if the presence of fibrillar HTTExon1Q25 and Q48 could alter the staining pattern of endogenous HTT. To do this, we evaluated signal intensity and aggregation in multiple brain regions and found that both measures were impacted by administration of Q48 fibrils. HTT signal intensity was significantly decreased in Q48-treated mice in the hippocampus, cortex and striatum (Treatment hippocampus: *F*_2,22_ = 7.283, *p* < 0.0037; treatment cortex: *F*_2,24_ = 7.633, *p* < 0.01; treatment striatum: *F*_2,25_ = 9.435, *p* < 0.001) (Online Resource 6c, d and e). In the hippocampus, WT mice injected with HTTExon1Q48 depicted a strong trend towards a decrease compared to HTTExon1Q25-treated mice (*p* = 0.0529) (Online Resource 6b and c), while R6/2 mice injected with HTTExon1Q48 displayed a significant reduction in signal intensity compared to BSA treated mice and a strong trend towards a decrease compared to Q25-treated mice (*p* = 0.0586) in the hippocampus (Online Resource 6c). In the cortex, no treatment differences were observed in WT mice but Q48-treated R6/2 mice had significantly reduced signal compared to both Q25- and BSA-treated mice (Online Resource 6d). HTTExon1Q48-treated WT and R6/2 mice displayed significantly decreased endogenous HTT signal intensity as compared to BSA mice (Online Resource 6e). In this region, the Q25 signal levels did not differ from either BSA- or Q48-treated animals. WT HTT aggregates were quantified in WT and R6/2 mice treated with Q25 and Q48 fibrils; however, aggregates were only detected in WT and R6/2 mice treated with Q48 (Online Resource 6f). These aggregates were observed throughout the brains (Online Resource 6g). Together, these changes in WT HTT staining provide strong evidence for an alteration of the WT protein distribution by Q48 fibrils.

### Peripheral injection of mHTTExon1 fibrils initiates an immune response

Both intracerebral and intraventricular infusion of exogenous mHTT fibrils is sufficient to induce a behavioral phenotype and a redistribution of endogenous HTT. To determine whether the behavioral phenotypes we observed upon injection of exogenous fibrillar HTTExon1 are due to their administration within the central nervous system, WT mice were injected intravenously with HTTExon1Q25 and Q48 fibrils every 2 weeks for 3 months (Online Resource 7a). Mice underwent a battery of behavioral tests each month to assess motor performance, cognition and anxiety-like behavior. Motor performance was assessed using distance traveled in the open field (Online Resource 7b), cognition was assessed by intersession habituation in the open field (Online Resource 7c) and spontaneous alternation in the Y-maze (Online Resource 7d). Anxiety-like behavior was measured using the light–dark box (Online Resource 7e). No behavioral changes were observed with any test at any time point. The absence of phenotypic changes may be explained by an increased immune response that was observed in mice injected with HTTExon1Q48 fibrils (Online Resource 7f).

## Discussion

We utilized multiple cellular and animal models to demonstrate that exogenous fibrillar mHTTExon1 is taken up, transported and seeds pathology within the brain in a manner similar to prions. We established that multiple cell types take up toxic HTTExon1 fibrils. Uptake is accompanied by the aggregation of endogenous HTT in SH-SY5Y cells, THP1-derived macrophages and iGABA human neurons derived from induced pluripotent stem cells. While all three cell models displayed many common features, there were also some notable differences. Firstly, each cell line incorporated fibrils to different extents over different time scales. iGABA cells took up exogenous fibrillar HTTExon1Q25 or Q48 to the least extent (with only approximately 0.3% of cells containing puncta after 3 days of exposure). This figure was 5% and 20% for SH-SY5Y and THP1-derived macrophages exposed to HTTExon1Q25 or Q48, respectively. The similarity between THP1 and SH-SY5Y cells, as compared to iGABA, suggests that higher uptake may be a feature of mitotic cell lines, in agreement with previous reports [46], although we did observe the phenomenon in THP1 cells which are also post-mitotic. The reason why cells exposed to HTTExon1Q25 or Q48 do not contain the same number of puncta may be due to a better processing of HTTExon1Q25 compared to HTTExon1Q48. Indeed, we previously showed, using Fourier-transform infrared spectroscopy (FTIR) spectroscopy, that the two fibrillar assemblies exhibit structural differences [38].

Although HTTExon1Q25 and Q48 fibrils were taken up by iGABA cells to a similar extent, higher stress levels were observed upon fibrillar HTTExon1Q48 uptake. Interestingly, the two neuron-like cells exhibited 1 or 2 puncta upon exposure to fibrillar HTTExon1, while macrophages had 30  or more much smaller puncta than those in neuronal cells. It is perhaps not overly surprising that a macrophage cell line attempts to degrade exogenous proteins to a greater degree than do neuronal cell lines. What is more striking, however, is that despite the differences in the processing of the fibrils, both neuronal and macrophage cell lines displayed increased aggregation of endogenous HTT, although at a much faster rate in macrophages. It is possible that the faster uptake kinetics and increased ability to degrade fibrils that characterizes macrophages also favor the interaction of endogenous and exogenous HTT and seeding. Regardless, the filter retardation assay (refer to Fig. 2) provides the first evidence that exogenous HTTExon1 fibrils seed the aggregation of endogenous HTT. Previous reports demonstrated seeding in transfected cells expressing high levels of endogenous HTT [38, 45, 46].

To determine whether what we observed in vitro applies to more complex systems, adult WT mice received intracortical injection of exogenous fibrillar HTTExon1. A single 2-μg dose of exogenous fibrils was sufficient to trigger a behavioral phenotype. However, the induction of a phenotype required a longer period of time than was previously shown after injection of iPSCs expressing mHTT [27]. Aside from the nature of HTT, there were other important factors that differed in the two protocols such as the age of the animals and site of administration [1].

A particularly salient finding in the WT adult background is the presence of behavioral and biochemical changes that persist beyond the presence of fibrils. Indeed, by 16 months of age, mice still displayed increased anxiety-like behavior, impaired cognition, and changes in the staining pattern of endogenous HTT even though fibrils were no longer detectable. This indicates a sustainable and irreversible change triggered by HTTExon1 fibrils. The decreased endogenous HTT immunodetection suggests that the persistent change caused by HTTExon1 fibrils may involve endogenous HTT. Interestingly, the changes in endogenous HTT state were most apparent in the vicinity of the cortical injection even though the striatum is the most affected region in HD. Although the regions with significant changes in endogenous HTT levels did not match with the pattern or regional susceptibility typical of HD pathology, the behavioral changes are consistent with typical disease manifestation. While the motor symptoms are the hallmark of HD and are necessary for the diagnosis of disease onset, they are not generally the first symptoms that present. In most patients and animal models of HD, cognitive deficits and anxiety-like behavior precede the onset of motor symptoms [18, 55]. It is therefore probable that at later stages, a motor phenotype may have begun to emerge. It is also possible that the motor phenotype was less evident at late time points due to the advanced age of the mice and the decrease in motor acuity that accrues as part of the aging process [2].

Studies in R6/2 mice further demonstrated the capacity of fibrillar HTTExon1Q48 to precipitate disease phenotype. In this second set of experiments, we included BSA as a negative control to determine if the injection of Q25 had any behavioral consequences. This control revealed that there were no significant differences between BSA-injected and HTTExonQ25-injected WT and R6/2 mice except for intrasession habituation at 4 weeks of age. Given that this difference was present only at 4 weeks, it appears to be more consistent with an exacerbated immune response to the presence of BSA than to a beneficial effect of Q25 on short-term memory performance. This is supported by previously published reports of the immunogenic properties of bovine serum albumin to the developing mouse brain [25]. The fact that cognitive tests at 4 weeks of age were most impacted is also consistent with this interpretation as it matches the pattern of behavioral abnormalities observed one month after injection of LPS, which is well known to induce acute neuroinflammation [48]. To support this hypothesis, we counted the number of microglia present around the site of injection 1 h post-surgery. At this time point, we did not observe any difference in immune activation between groups. This does not necessarily exclude an immune response as LPS-induced inflammatory effects require more than 1 h to reach a maximal effect in vitro [11] and experiments generally focus on 24 h post-injection in vivo [48, 58] although changes in Iba-1 expression have been observed as early as 2 h [48]. Given the focus on later time points with the very immunogenic nature of LPS, it is not surprising that an immune response is not observed 1 h post-injection.

Aside from supporting a detrimental effect of HTTExon1Q48, this experiment also highlighted the importance of a number of factors. The ventricular injection site, injection volume (bilateral 2 µg injection vs. unilateral 2 µg injection) and age led to a much more rapid onset of phenotype in the R6/2 paradigm with more apparent aggregation of WT HTT than in the adult WT paradigm. At 12 weeks of age, changes in behavior and endogenous HTT state were present in WT injected pups that did not arise until 12 or 16 months of age in injected adult WT and aggregation of WT HTT was observed in both R6/2 and WT mice, which was not observed at any time point in adult WT mice. This raises interesting questions regarding both the role of HTT in development as well as the role of development in prion protein spreading, in general. The increased concentration of fibrils may also have played a role, but it is unlikely that the two-fold increase when coupled with a change in site of injection could explain the 9-month difference in induction time that was observed between the two experiments.

To determine if central administration of fibrils is necessary to induce behavioral changes, WT mice received intravenous injection of fibrils. With this paradigm, no changes in behavior were observed at 3 months of age. This time point is early to expect a behavioral phenotype but the experiment was aborted because assessment of the blood revealed that peripheral infusion of HTT and mHTT fibrils resulted in an immune response. Interestingly, fibrillar HTTExon1Q48 induced the highest immune response that most probably effectively blocked exogenous fibrillar HTTExon1-mediated changes within the central nervous system.

The multiple models utilized in this study allow us to conclude that HTTExon1Q48 fibrils are taken up by cells, both in vitro and in vivo, and trigger cellular dysfunction and behavioral changes in cells and mice after uptake. These changes are also associated with changes in the state of endogenous HTT. Together, these results strongly suggest that at least certain forms of HTT are capable of spreading and seeding disease; in other words, HTT fibrils have prion-like properties. While the importance of prion-like spreading of mHTT within normal HD pathophysiology is still unknown, studies of healthy tissue grafts implanted into the brains of HD patients demonstrated that mHTT protein could be found in the grafted tissue originating from healthy individuals [12]. This grafted tissue was derived from healthy donors expressing HTT with non-pathogenic CAG expansions. Thus, the only possible source of mHTT was the surrounding tissue. These findings, together with our observations in R6/2 mice, suggest that spread may occur in the patients’ central nervous system, thus contributing to disease progression. There is a large degree of heterogeneity in the clinical features of HD [41] in patients with the same CAG repeat length, which implies that there are, as of yet, undescribed factors contributing to clinical presentation [22]. While these factors may have a genetic basis (e.g., tau and cognitive decline [21, 53], proteostasis and age of onset [20, 29]), our data indicate that non-cell autonomous spread of mHTT is a novel modifier of HD disease progression.

## Electronic supplementary material

Below is the link to the electronic supplementary material.

Supplementary material 1 (TIFF 29572 kb)

Supplementary material 2 (TIFF 4868 kb)

Supplementary material 3 (TIFF 10471 kb)

Supplementary material 4 (TIFF 28953 kb)

Supplementary material 5 (TIFF 17965 kb)

Supplementary material 6 (TIFF 21859 kb)

Supplementary material 7 (TIFF 33895 kb)

Supplementary material 8 (DOC 22 kb)
