# Supplementary figures and images for: Demonstration of prion-like properties of mutant huntingtin fibrils in both in vitro and in vivo paradigms
Source: Acta Neuropathol. 2019 Feb 20;137(6):981–1001. doi: 10.1007/s00401-019-01973-6 (PMC6531424; doi:10.1007/s00401-019-01973-6)

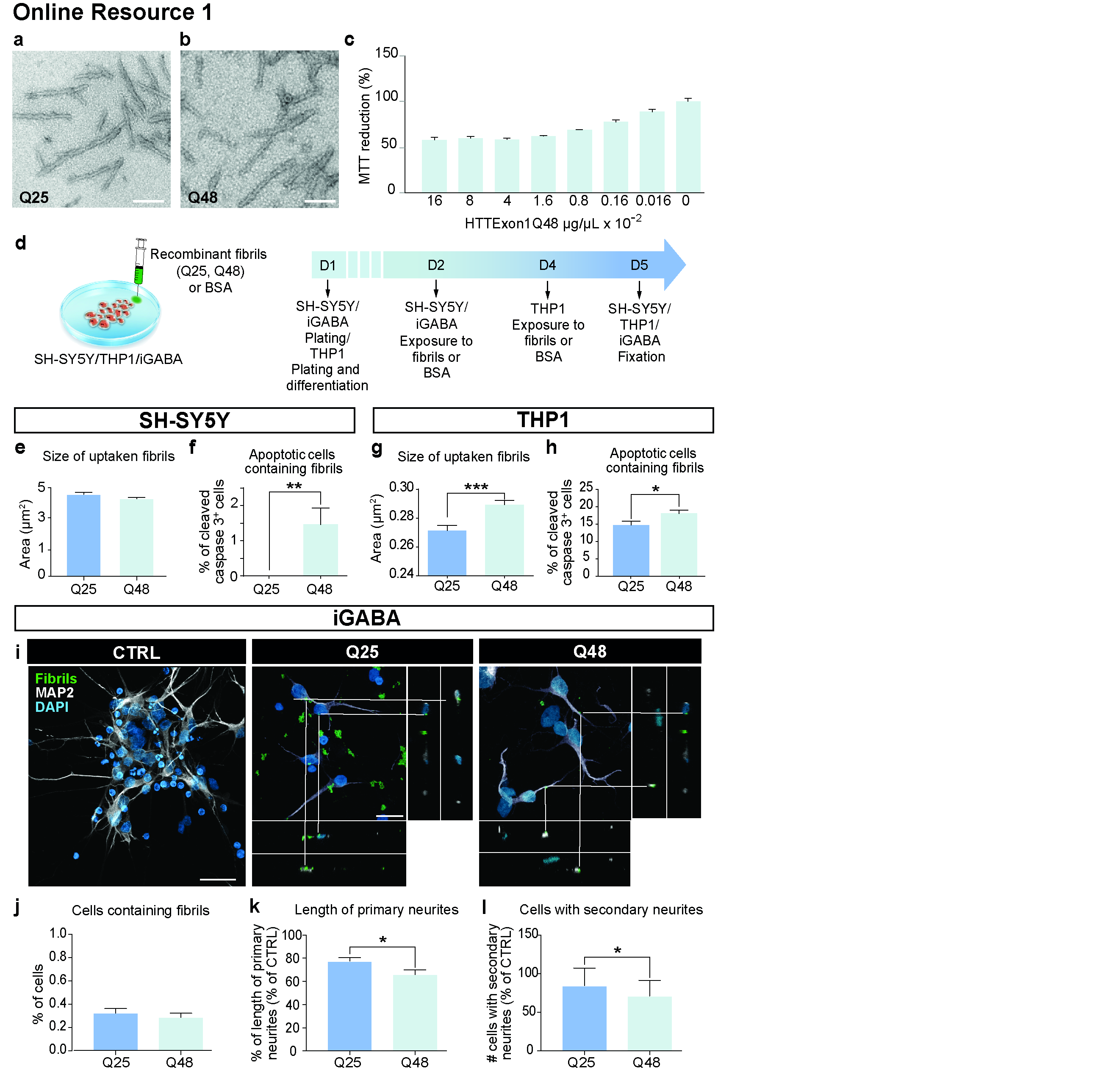

Supplement: Supplementary file 1 — Supplementary material 1 (TIFF 29572 kb) [file 401_2019_1973_MOESM1_ESM.tif]

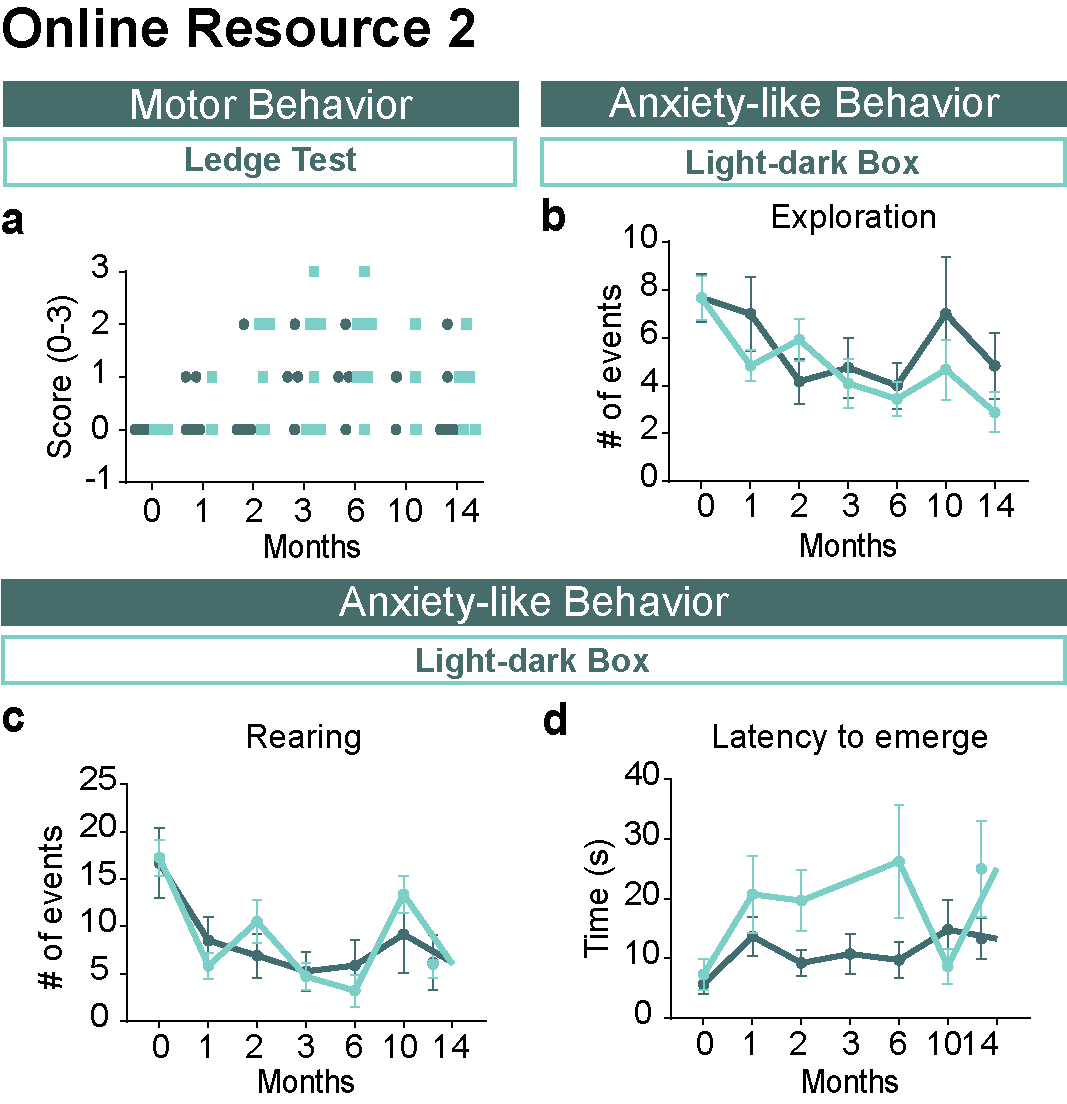

Supplement: Supplementary file 2 — Supplementary material 2 (TIFF 4868 kb) [file 401_2019_1973_MOESM2_ESM.tif]

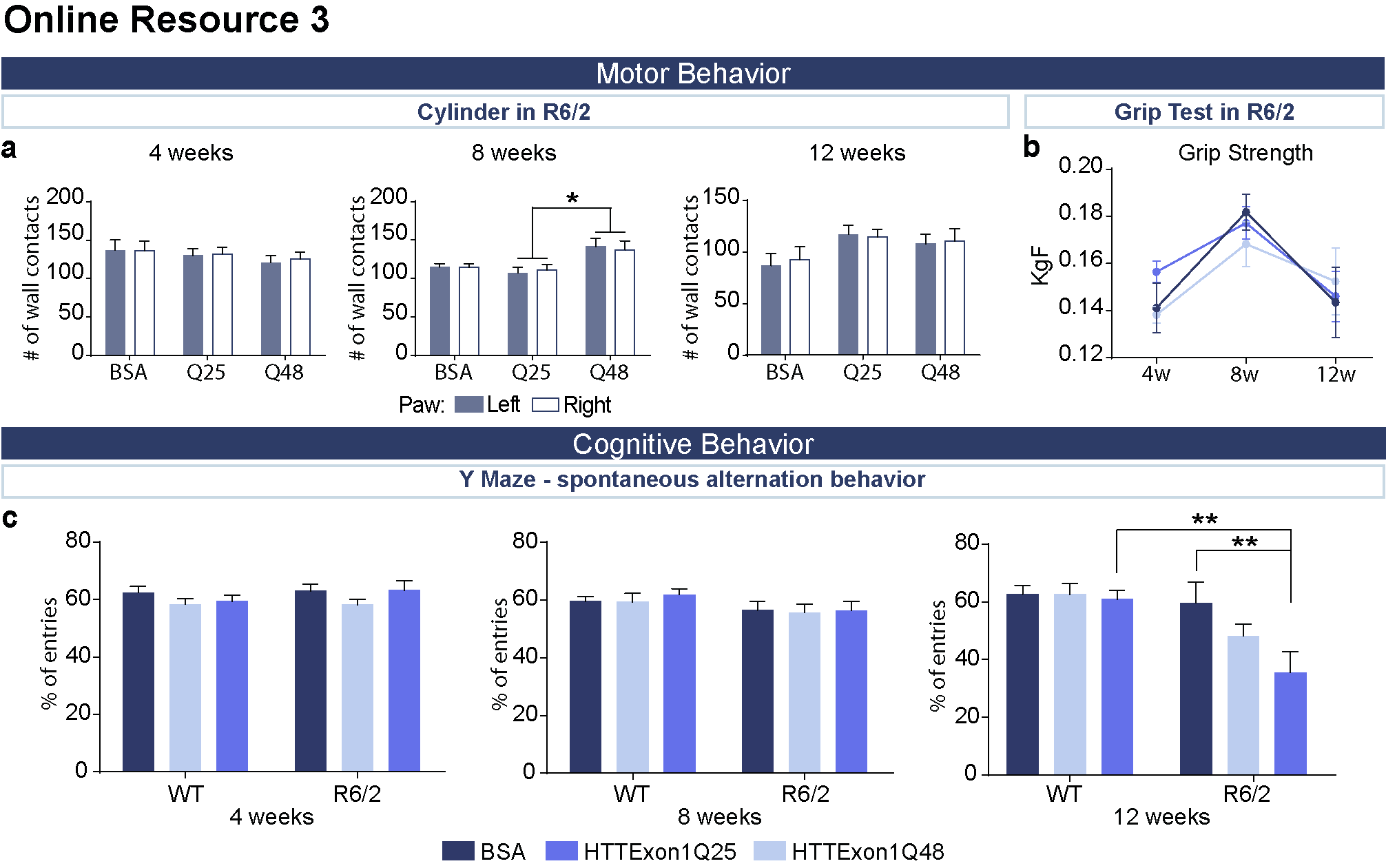

Supplement: Supplementary file 3 — Supplementary material 3 (TIFF 10471 kb) [file 401_2019_1973_MOESM3_ESM.tif]

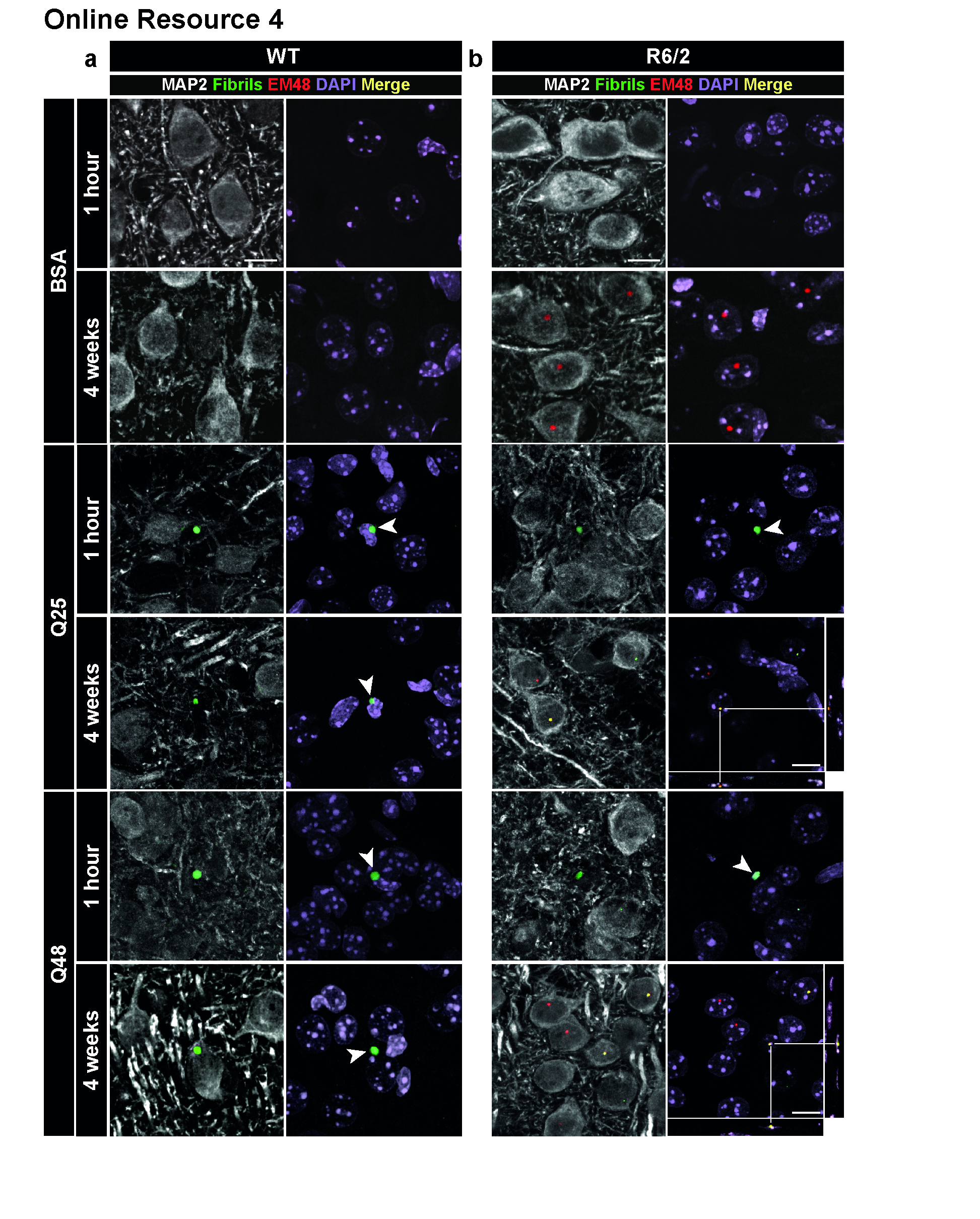

Supplement: Supplementary file 4 — Supplementary material 4 (TIFF 28953 kb) [file 401_2019_1973_MOESM4_ESM.tif]

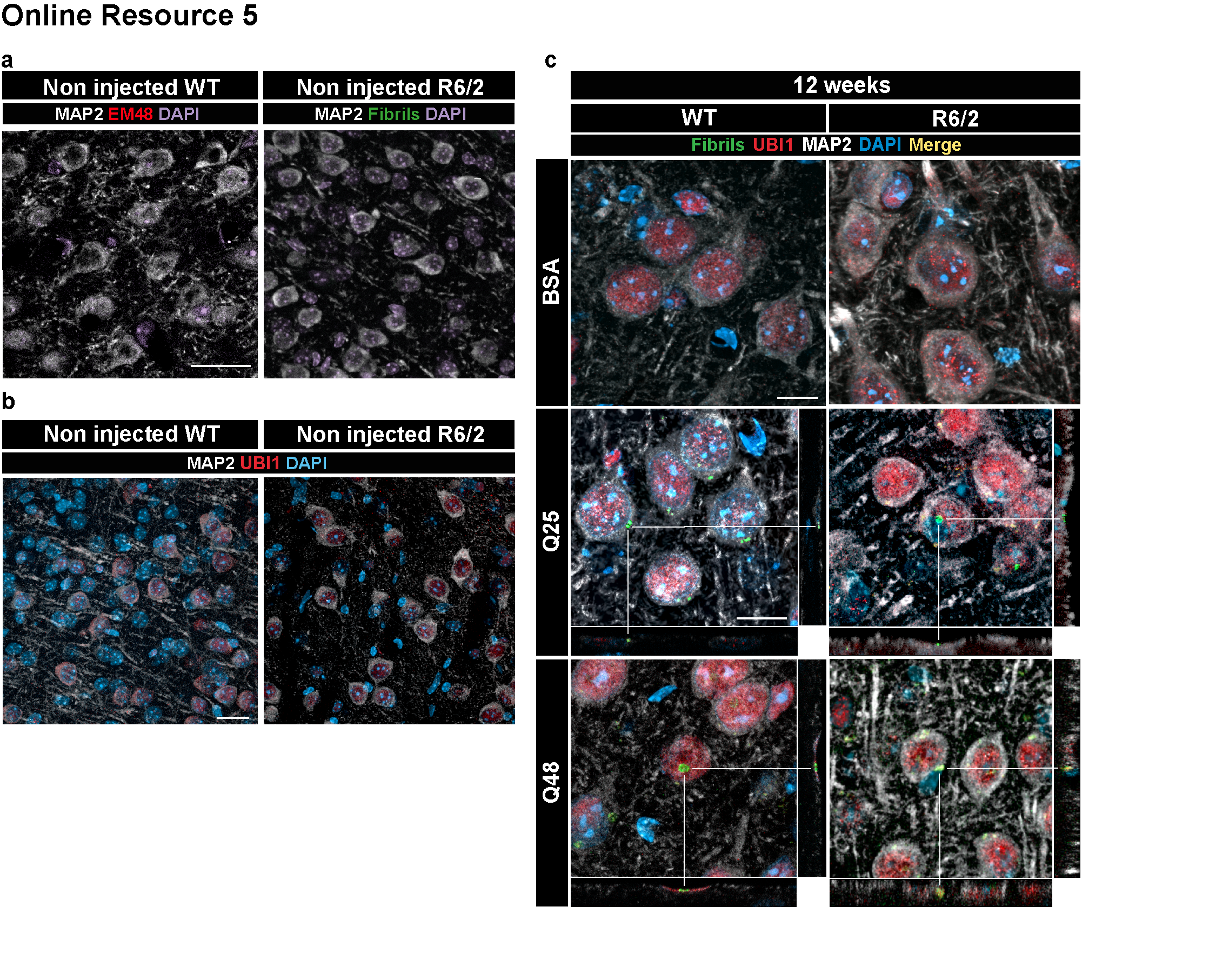

Supplement: Supplementary file 5 — Supplementary material 5 (TIFF 17965 kb) [file 401_2019_1973_MOESM5_ESM.tif]

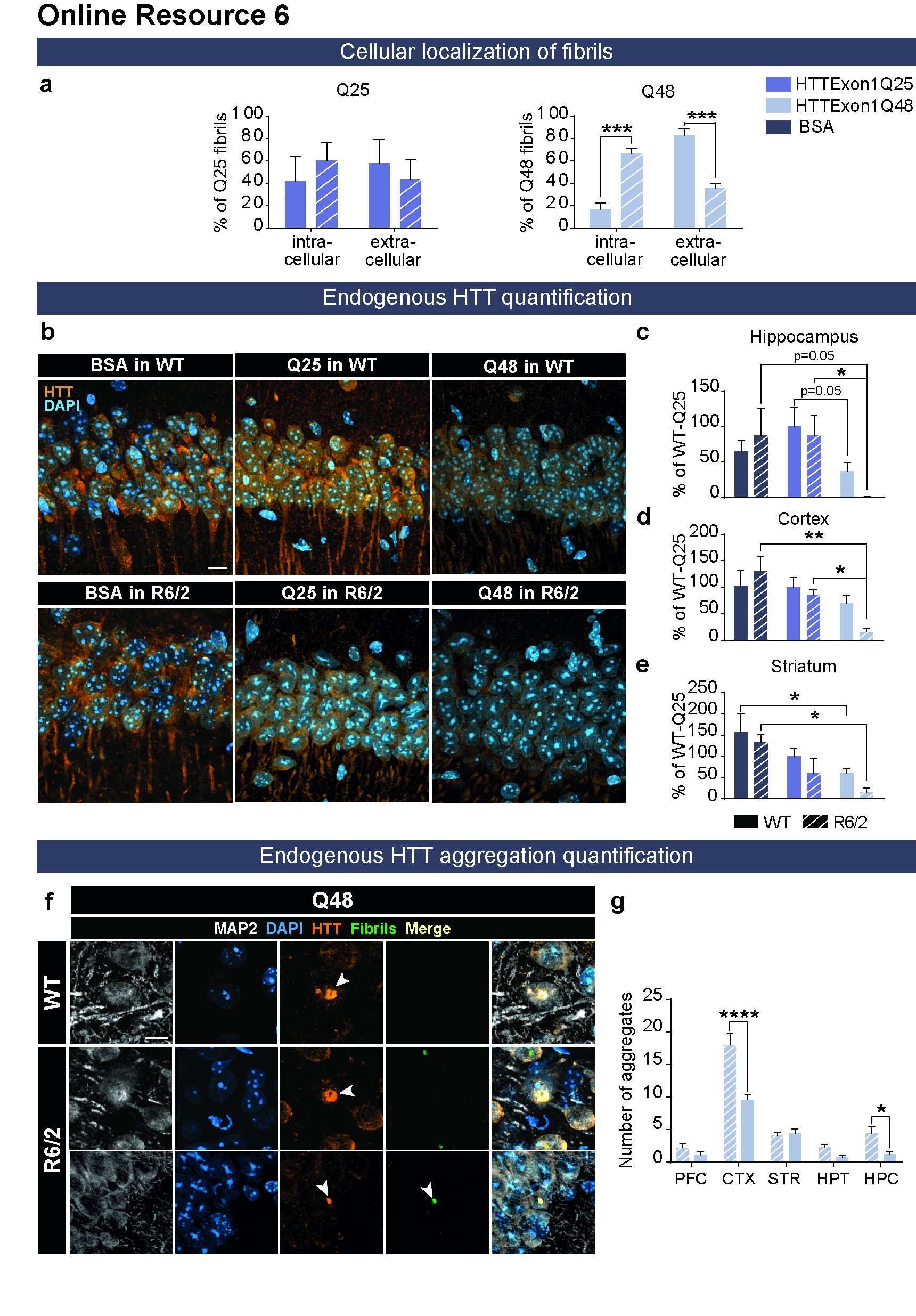

Supplement: Supplementary file 6 — Supplementary material 6 (TIFF 21859 kb) [file 401_2019_1973_MOESM6_ESM.tif]

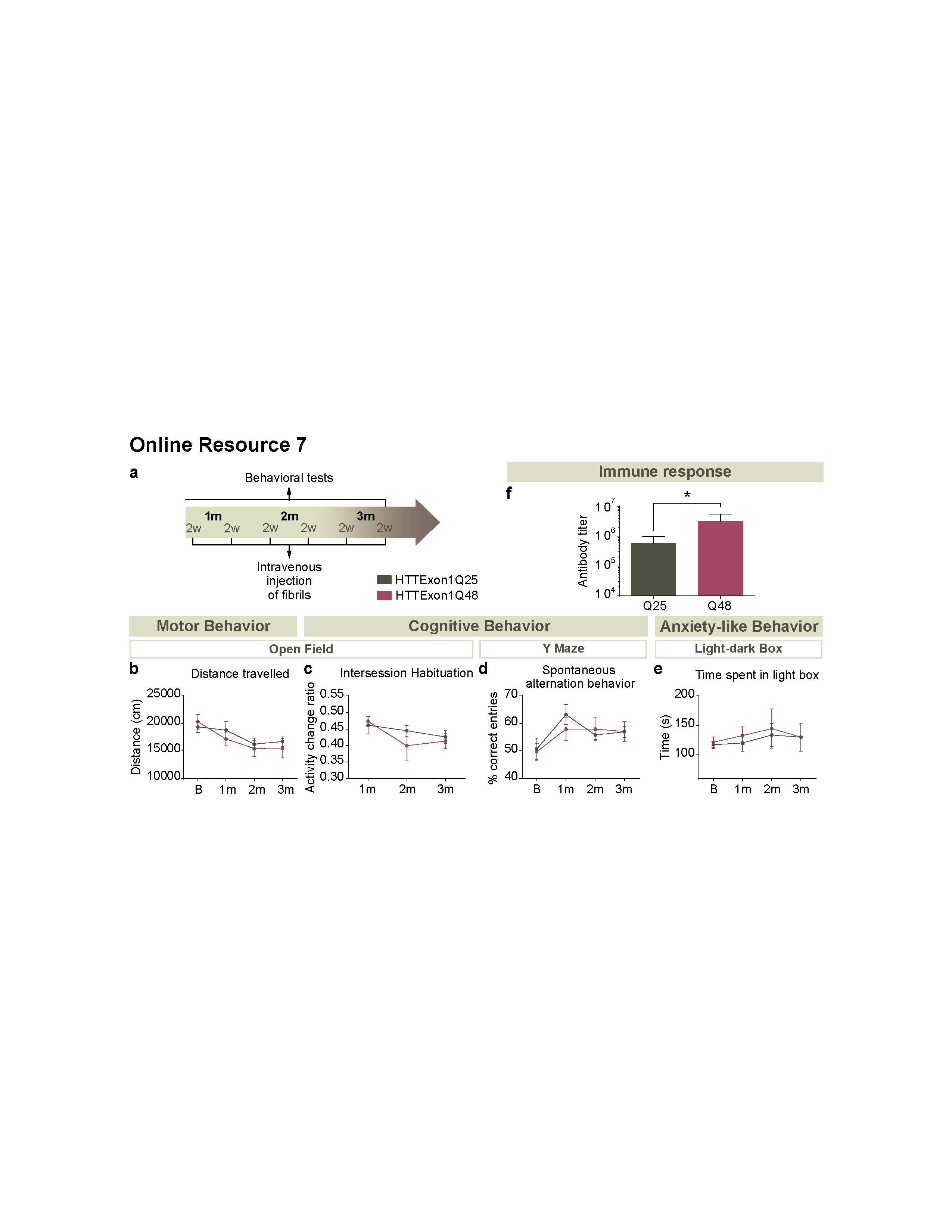

Supplement: Supplementary file 7 — Supplementary material 7 (TIFF 33895 kb) [file 401_2019_1973_MOESM7_ESM.tif]
