# Supplementary material for: Demonstration of prion-like properties of mutant huntingtin fibrils in both in vitro and in vivo paradigms
Source: Acta Neuropathol. 2019 Feb 20;137(6):981–1001. doi: 10.1007/s00401-019-01973-6 (PMC6531424; doi:10.1007/s00401-019-01973-6)
Supplement: Supplementary file 8 — Supplementary material 8 (DOC 22 kb) [file 401_2019_1973_MOESM8_ESM.docx]

**Supplementary materials**

**Demonstration of prion-like properties of mutant huntingtin fibrils in both *in vitro* and *in vivo* paradigms**

**Acta Neuropathologica**

Maria Masnata*^1,2^, Giacomo Sciacca*^1,2^, Alexander Maxan*^1,2^, Luc Bousset*^4^, Hélѐna Denis^1,2^, Florian Lauruol^1,2^, Linda David^1,2^, Martine Saint-Pierre^1,2^, Jeffrey H. Kordower^3^, Ronald Melki^^4^, Melanie Alpaugh^^1,2^, Francesca Cicchetti^^1, 2^

^1^Centre de Recherche du CHU de Québec, Axe Neurosciences, 2705 Boulevard Laurier, Québec, QC, Canada; ^2^Département de Psychiatrie & Neurosciences, Université Laval, Québec, QC, Canada; ^3^Department of Neurological Sciences, Rush University Medical Centre, Chicago, Illinois, USA;

^4^Laboratory of Neurodegenerative Diseases, Institut François Jacob, MIRCen, CEA, CNRS, Fontenay-aux-Roses, France

**Equal contribution; ^Co-corresponding authors*

**Correspondence to either:**

Ronald Melki, Ph.D., Institut François Jacob (MIRCen), Email: [ronald.melki@cnrs.fr](mailto:ronald.melki@cnrs.fr)

Melanie Alpaugh, Ph.D., Centre de Recherche du CHU de Québec, Email: [melanie-jeanne.alpaugh.1@ulaval.ca](mailto:melanie-jeanne.alpaugh.1@ulaval.ca)

Francesca Cicchetti, Ph.D., Centre de Recherche du CHU de Québec, Email: [francesca.cicchetti@crchul.ulaval.ca](mailto:francesca.cicchetti@crchul.ulaval.ca)

**SUPPLEMENTAL FIGURE LEGENDS**

**Online Resource 1. Toxic effects of exogenous HTTExon1Q48 fibrils on multiple cell lines.** Representative electron microscopy images of HTTExon1Q25 (**a**) and Q48 fibrils (**b**). Dose response curve showing reduction in MTT levels after exposure to increasing concentrations of HTTExon1Q48 fibrils (**c**). Experimental design and timeline of treatment with ATTO488-labeled HTTExon1Q25 and Q48 fibrils (**d**). The size of HTTExon1Q25 and 48 fibrils within SH-SY5Y (**e**) and THP1 cells (**g**) were measured. The population of cells containing fibrils was isolated and within this population, an increase in the percentage of caspase 3+ cells was detected in both SH-SY5Y (**f**) and THP1 (**h**) cells after exposure to HTTExon1Q48 fibrils. In THP1 cells, the size of intracellular aggregates (**g**) and the number of caspase 3+ cells containing fibrils (**h**) were also quantified. The percentage of caspase 3+ cells in HTTExon1Q25 and HTTExon1Q48 cells was calculated compared to BSA control (**h**). iGABA neurons demonstrate uptake of both HTTExon1Q25 and HTTExon1Q48 fibrils after treatment. Immunostaining against microtubule-associated protein MAP2 (white) and DAPI staining to mark the nuclei (blue) were performed, while fibrils labeled with ATTO-488 (**i**). The number of cells with puncta were counted (**j**). The effect of fibrils on morphology was assessed by measuring the length each cell’s primary neurite (**k**) and the number of neurons with secondary projections (**l**). Scale bars **a and b** = 100 nm; **i** = 10 µm. Data are expressed as mean +/- SEM. Statistical analysis was performed using student’s unpaired t-test. All graphs are the average of three independent replicates. *p<0.05, ** p<0.01. *BSA* bovine serum albumin, *CTRL* control, *MAP2* microtubule associated protein

**Online Resource 2. Absence of motor impairments in WT adult mice injected with HTTExon1Q48 fibrils.** Motor performance was characterized using the ledge test (**a**). Motor confound were absent from the light-dark box as measured by exploration (**b**) and rearing (**c**). Anxiety-like behavior by analysis of latency to emerge head from the dark box in the light-dark box test (**d**). Data are expressed as mean +/- SEM. HttExon1Q25 *n*=9-12, HTTExon1Q48 *n*=9-12. Statistical analysis was calculated using a repeated measures two-way ANOVA with Tukey’s post-hoc tests

**Online Resource 3. Examples of behavioral measures not affected in R6/2 mice following injection of HTTExon1Q48 fibrils.** Motor performance in R6/2 mice was also assessed using the cylinder task (R6/2 BSA *n*=9-16; R6/2 HTTExon1Q25 *n*=12-17; R6/2 HTTExon1Q48 *n*=9-18) (**a**) and grip test (R6/2 BSA *n*=9; R6/2 HTTExon1Q25 *n*=13; R6/2 HTTExon1Q48 *n*=9) (**b**). Cognitive performance was measured at 4, 8 and 12 weeks of age using the Y-maze (WT BSA *n*=12-13; WT HTTExon1Q25 *n*=10-13; WT HTTExon1Q48 *n*=10-14; R6/2 BSA *n*=8-16; R6/2 HTTExon1Q25 *n*=12-15; R6/2 HTTExon1Q48 *n*=9-18) (**c**). Data are expressed as mean +/- SEM. Statistical analysis was performed using a two-way ANOVA with Tukey’s post-hoc tests except for **b** where a repeated measures two-way ANOVA was performed. *p<0.05. *BSA* bovine serum albumin*, WT* wild type

**Online Resource 4. Colocalization of HTTExon1Q48 fibrils with endogenous mHTT in R6/2 mice at 4 weeks of age.** Fibrillar puncta were detected in WT (**a**) and R6/2 mice brains (**b**) at 1 h and 4 weeks post-injection of HTTExon1Q25 and Q48, but not BSA. Arrowheads indicate puncta. Quadruple immunofluorescence for fibrils (green), mHTT aggregates EM48 (red), MAP2 (white) and cell nuclei DAPI (purple). Merged EM48 and fibrillar puncta is visualized with yellow. Scale bars: 10 µm. *BSA* bovine serum albumin *MAP2* microtubule associated protein 2, *WT* wild type

**Online Resource 5. Colocalization of HTTExon1Q48 fibrils with ubiquitin.** The specificity of EM48 (**a**) and ubiquitin (UBI1) (**b**) was confirmed in non injected WT and R6/2 mice. To further support this, UBI1 staining was used as an additional marker of endogenous mHTT. It did not colocalize with fibrils in WT mice, but in R6/2 mice (**c**), particularly R6/2 mice injected with HTTExon1Q48 fibrils at 12 weeks post-injection. Quadruple immunofluorescence for fibrils (green), mHTT aggregates by either ubiquitin or EM48 (red), MAP2 (white) and cell nuclei DAPI (purple/blue). Scale bar **a, b** = 10 µm; **c**= 20 µm. *MAP2* microtubule associated protein 2, *WT* wild type

**Online Resource 6. Changes in staining patterns of endogenous HTT following injection of HTTExon1Q48 fibrils.** The presence of fibrillar puncta inside and outside the cells was quantified for HTTExon1Q25 and Q48 fibrils in WT and R6/2 mice (**a**). WT HTTExon1Q25 *n*=5, WT HTTExon1Q48 *n*=5, R6/2 HTTExon1Q25 *n*=5, R6/2 HTTExon1Q48 *n*=5. Representative confocal photomicrographs of endogenous HTT immunoreactivity in the hippocampus of WT and R6/2 mice (**b**) and quantification of staining intensity for hippocampus (**c**), cortex (**d**) and striatum (**e**). Quantification data is shown as percentage of area stained for Q25-treated WT controls. WT BSA *n*=5; WT HTTExon1Q25 *n*=6; WT HTTExon1Q48 *n*=6, R6/2 BSA *n*=3, R6/2 HTTExon1Q25 *n*=2-4, R6/2 HTTExon1Q48 *n*=6. Representative confocal photomicrographs depicting changes in the staining pattern of endogenous HTT in the animals injected with Q48 (**f**) and quantification of the number of endogenous HTT aggregates detected in different brain regions (**g**). WT HTTExon1Q25 *n*=10, WT HTTExon1Q48 *n*=10, R6/2 HTTExon1Q25 *n*=10, R6/2 HTTExon1Q48 *n*=10. Data are expressed as mean +/- SEM. Statistical analysis was performed using a two-way ANOVA with Tukey’s post-hoc tests. *p<0.05, *** p<0.001. Scale bar = 10 µm. *BSA* bovine serum albumin, *HTT* huntingtin, *WT* wild type

**Online Resource 7. Development of an immune response following intravenous injection of HTTExon1Q25 and Q48 fibrils in adult WT mice.** Experimental timeline (**a**). Motor performance was assessed using distance travelled in the first 5 min in the open field (**b**). Long-term memory was measured by the change in distance travelled in the first 5 min of each testing time point (**c**). Working memory was measured by spontaneous alternations in the Y-maze (**d**). Anxiety-like behavior was measured by time spent in the light box of the light-dark box (**e**). Blood was collected at the end of the experiment and the antibody titre in HTTExon1Q25 and Q48 injected mice was assessed (**f**). Data are expressed as mean +/- SEM. WT HTTExon1Q25 *n*=8, WT HTTExon1Q48 *n*=7. Statistical analysis was performed using a repeated measures two-way ANOVA with Tukey’s post-hoc tests except for **f** where a student’s unpaired t-test was utilized. *p<0.05. *B* baseline, *BSA* bovine serum albumin, *m* month, *w* week, *WT* wild type
